# Corticotropin-releasing factor neurons in the bed nucleus of the stria terminalis modulate avoidance behaviors and feeding

**DOI:** 10.3389/fnins.2026.1876369

**Published:** 2026-07-20

**Authors:** Juan Manuel Romero, Shae-Marie Stafford-Trujillo, Mitzy Mendoza, Pey-Shyuan Chin, Snigdha Srivastava, Mikhail Kochukov, Qingchun Tong, Benjamin Russell Arenkiel

**Affiliations:** 1 Department of Neuroscience, Baylor College of Medicine, Houston, TX, United States; 2 Department of Molecular and Human Genetics, Baylor College of Medicine, Houston, TX, United States; 3 Medical Scientist Training Program, Baylor College of Medicine, Houston, TX, United States; 4 Jan and Dan Duncan Neurological Research Institute, Baylor College of Medicine, Houston, TX, United States; 5 Brown Institute of Molecular Medicine at McGovern Medical School, University of Texas Health Science Center of Houston, Houston, TX, United States; 6 Neuroscience Program at UT MD Anderson Cancer Center UTHealth Houston Graduate School of Biomedical Sciences, University of Texas Health Science Center of Houston, Houston, TX, United States

**Keywords:** avoidance behavior, bed nucleus of the stria terminalis, corticotropin-releasing factor, feeding behavior, hypothalamus, mouse

## Abstract

**Introduction:**

Animals rely on defensive avoidance of exposed or potentially threatening environments as a fundamental survival strategy, often expressing this behavior as thigmotaxis, or a preference for the periphery of an open space. However, researchers still do not understand how neural circuits coordinate avoidance behavior with competing physiological drives such as feeding. In this study, we investigate how corticotropin-releasing factor (CRF)-expressing neurons in the bed nucleus of the stria terminalis (BNST) integrate environmental avoidance and feeding behavior.

**Methods:**

We used adult male and female mice and applied fiber photometry to monitor BNST CRF neuronal activity in vivo. We performed both acute and chronic manipulations of these neurons to test their causal role in behavior. We used behavioral assays to measure avoidance (thigmotaxis) and food intake. We also conducted anatomical tracing to identify projections from BNST CRF neurons to hypothalamic regions and used axon terminal optogenetic stimulation to test the functional contributions of specific pathways.

**Results:**

BNST CRF neurons increased their activity in response to diverse stressors. When we activated these neurons, mice showed increased periphery-oriented behavior and reduced food intake. Anatomical tracing revealed that BNST CRF neurons project to key hypothalamic regions, including the lateral hypothalamus (LH) and paraventricular hypothalamic nucleus (PVH). When we stimulated BNST CRF→LH projections, we observed both increased avoidance behavior and suppressed feeding. In contrast, stimulating BNST CRF→PVH projections did not significantly alter either behavior.

**Discussion:**

Our findings identify BNST CRF neurons as a circuit node that integrates stress and feeding-related signals to bias behavioral output. Specifically, BNST CRF projections to the LH drive the coupling of avoidance and feeding suppression. These results provide new insight into how the brain coordinates competing motivational states such as avoidance and feeding.

## 1 Introduction

Avoidance is a fundamental behavioral strategy conserved across species, enabling organisms to minimize exposure to potential threats in their environment (López-Moraga et al., 2022; McManus and Milad, 2025; Tseng et al., 2023). One of the most ethologically relevant manifestations of avoidance is thigmotaxis — the tendency to remain close to physical boundaries or walls — which is widely observed in rodents and other animals as a defensive response to perceived danger (Kallai et al., 2007; Simon et al., 1994). This behavior reflects a broader class of threat-avoidance strategies that support survival. Importantly, such avoidance behaviors are not only adaptive in naturalistic contexts but also serve as behavioral readouts in models of neuropsychiatric disorders, including anxiety, depression, and feeding-related pathologies (Ironside et al., 2020; Sporrer et al., 2026).

At the neural level, avoidance behaviors are orchestrated by a distributed network of brain regions that include the hypothalamus, amygdala, prefrontal cortex, insular cortex, hippocampus, and the bed nucleus of the stria terminalis (BNST) (Hu et al., 2020; King et al., 2017). Among these, the BNST has emerged as a critical hub for integrating sensory, emotional, and homeostatic signals to guide behavioral responses to uncertain or ambiguous threats. Indeed, lesions of the BNST abolish anxiety-like responses to contexts of unpredictable threat, underscoring its necessity in mediating sustained avoidance states (Walker and Davis, 1997)

Anatomically, the BNST receives convergent input from stress-responsive regions such as the hypothalamus and emotionally salient structures like the amygdala, allowing it to compute the salience and valence of environmental cues (Sun et al., 2023). Its efferent projections to the lateral hypothalamus (LH), paraventricular hypothalamus (PVH) and amygdala enable modulation of both behavioral and physiological outputs. Notably, the BNST contains dense populations of corticotropin-releasing factor (CRF)-expressing neurons, which are sensitive to anxiogenic stimuli (Butler et al., 2016) and stress exposure (Hu et al., 2020). These CRF neurons represent a molecularly distinct subpopulation within the broader, heterogeneous BNST GABAergic network. In fact, GABAergic projections from the BNST to the LH have been shown to promote feeding (Jennings et al., 2013a). However, the extent to which these CRF BNST neurons coordinate avoidance behaviors with competing physiological demands, such as feeding, remains poorly understood.

Feeding behavior must be flexibly regulated to balance metabolic demand against competing behavioral priorities, including threat avoidance. Neural circuits controlling feeding integrate internal metabolic signals with external environmental cues, including those that signal threat or uncertainty (Kreifeldt et al., 2022; Stone and Brownell, 1994; Jovanovic et al., 2023). The CRF system, particularly within the hypothalamus, is known to modulate feeding during stress (Melnick et al., 2020; Carlin et al., 2006; Hill et al., 2013) suggesting that BNST CRF neurons may similarly influence feeding behavior in the context of threat-related avoidance.

In this study, we investigated the circuit-level mechanisms by which BNST CRF neurons regulate avoidance-related behaviors and feeding behavior. Using genetically targeted viral strategies and *in vivo* calcium imaging, we monitored the activity of BNST CRF neurons during exposure to varied avoidance driving stimuli. Gain-of-function manipulations revealed that activation of these neurons was sufficient to modulate both avoidance and feeding behaviors. These findings identify a BNST circuit that integrates ethologically relevant threat-avoidance signals with consummatory behavior to coordinate adaptive behavioral responses.

## 2 Materials and methods

### 2.1 Animal care

All animal procedures were conducted in accordance with Baylor College of Medicine Institutional Animal Care and Use Committee (IACUC) guidelines and were approved under protocol AN5596. Methods are reported in accordance with ARRIVE recommendations. Mice were housed under standard conditions on a 12 h light/12 h dark cycle with ad libitum access to water and standard chow (Harlan 2920X). CRH-Cre mice (Crh^∧^tm1(cre)Zjh/J, strain #012704; The Jackson Laboratory, hereafter referred to as CRF-Cre) were used for all experiments. Both male and female mice were included. Animals were at least 8 weeks of age at the time of stereotaxic surgery.

### 2.2 Stereotaxic viral injections

Mice were anesthetized with vaporized isoflurane in oxygen (1%–3% for induction and maintenance). Surgeries were performed using a stereotaxic frame interfaced with Angle Two software. The skull was leveled in mediolateral and anteroposterior axes (±0.02 mm). Targeting coordinates were empirically verified using control DiI injections. The bed nucleus of the stria terminalis (BNST) was injected bilaterally at the following coordinates relative to bregma: AP +0.3 mm, ML ± 0.5 mm, DV −4.3 mm.

For anatomical tracing, rAAV-Ef1α-flex-synaptophysin::GFP-WPRE-hGHpA (DJ8) was used. For acute optogenetic manipulations, rAAV-Ef1α-flex-hChR2(H134R)-EYFP-WPRE-hGHpA (DJ8) was used, with rAAV-Ef1α-flex-eGFP (DJ8) as control. For chronic behavioral and metabolic manipulations, rAAV-Ef1α-Flex-eGFP-p2a-mNaCh-WPRE-hGHpA (DJ8) was used, with rAAV-Ef1α-flex-eGFP (DJ8) as control. For calcium imaging, rAAV-Synapsin-flex-jGCaMP8s-WPRE-pA (DJ8) was used. All viral preparations were titered to ≥1 × 10^11^ viral particles/μL.

### 2.3 Immunofluorescence and microscopy

At least 2 weeks following viral injection, mice were anesthetized with isoflurane and transcardially perfused with PBS followed by 4% paraformaldehyde (PFA; diluted from 16% EM grade PFA, Electron Microscopy Sciences). Brains were dissected and post-fixed overnight in 4% PFA at 4 °C, cryoprotected sequentially in 20% and 30% sucrose, embedded in O.C.T. compound, and stored at −80 °C. Coronal sections (40 μm) were cut using a cryostat (Leica CM1860), and every other section was collected.

For GFP immunofluorescence, free-floating sections were blocked in 10% horse serum in PBS-T (1 × PBS, 1% Triton X-100) for 1 h at room temperature, incubated overnight at 4 °C with goat anti-GFP primary antibody (1:300; Abcam AB6673), washed, and incubated with 1:1000 donkey anti-goat Alexa Fluor 647 secondary antibody (Thermofisher) for 2 h at room temperature. Sections were mounted using DAPI Fluoromount-G. Imaging was performed using Leica TCS SP8 STED or SP8X microscopes, with 10 × objectives for global images and 20 × objectives for insets.

To validate BNST CRF neuron projections to the lateral hypothalamus (LH) and paraventricular hypothalamic nucleus (PVH), rAAV-Ef1α-FLEX-synaptophysin::GFP-WPRE-hGHpA was injected into the BNST of two male and two female CRF-Cre mice. Two weeks later, brains were processed as described above and examined for GFP-labeled axonal fibers. Analyses were restricted to predefined hypothalamic regions of interest based on prior connectivity data from the Allen Mouse Brain Connectivity Atlas (Projection Experiment Page::Allen Brain Atlas: Mouse Connectivity and Projection Experiment Page::Allen Brain Atlas: Mouse Connectivity). Projection presence was assessed qualitatively rather than via whole-brain quantification.

### 2.4 Fiber photometry

#### 2.4.1 Implantation and recording

For BNST CRF calcium recordings, CRF- Cre mice were unilaterally injected in the BNST with rAAV-Syn-FLEX-jGCaMP8s. Immediately after injection, a fiber optic implant (200 μm core, NA 0.50; RWD R-FOC-L200C-50NA, RWD Life Science, Shenzhen, China) was placed above the left BNST (AP +0.3 mm, ML −0.5 mm, DV −3.9 mm) and secured with Metabond dental cement. Animals recovered and expressed virus for 5 weeks prior to recording.

Mice were habituated to patch- cord attachment for ≥5 days before testing, each day for 15 min. On test days, animals were connected in the home cage and acclimated for 15 min. A baseline recording was collected before behavioral testing. Photometry was recorded throughout each behavioral session and during post- stimulus periods when applicable.

#### 2.4.1 Photometry pre-processing and normalization:

Photometry signals were analyzed using custom Python scripts. Prior to all analysis, raw fluorescence signals from both the 405 nm isosbestic control channel and the 465 nm calcium-dependent channel were passed through a zero-phase second-order Butterworth low-pass filter with a 2 Hz cutoff to attenuate high-frequency noise while preserving slower calcium dynamics. All subsequent analysis, including regression fitting, ΔF/F computation, and statistical testing, were performed on these filtered signals. As all analyses were performed on 2 Hz low-pass filtered signals, traces presented in all figures reflect these filtered ΔF/F signals.

For each animal, a linear regression model was fit between the 405 nm isosbestic [F_405_(t)] and 465 nm calcium-dependent signals [F_465_(t)] during the pre-stimulus or pre-event baseline (as described in each relevant experiment):


$${\hat{F}}_{465}\,\left(t\right)=\alpha \times F_{405}\,\left(t\right)+\beta$$

where α and β are the slope and intercept, respectively, estimated from the baseline period. This fitted model for ${\hat{F}}_{465}\,\left(t\right)$ was applied to the 405 nm signal across the full recording session to generate a session-specific reference signal that captures variance attributable to motion and photobleaching. Regression-corrected ΔF/F was then computed as:


$$\frac{\Delta \,F_{465}}{{\hat{F}}_{465}\,\left(t\right)}=\frac{\left(F_{465}\,\left(t\right)-{\hat{F}}_{465}\,\left(t\right)\right)}{{\hat{F}}_{465}\,\left(t\right)}$$

where the numerator represents the calcium-dependent signal component that *cannot* be attributed to motion or photobleaching. Raw 405 nm isosbestic traces are provided as a supplement to permit independent verification of signal stability across sessions.

#### 2.4.2 Forced swim test and predator odor analysis

Recordings included a 60-s pre-stimulus baseline period (as described above), the stimulus period (forced swim or predator odor exposure), and a post- stimulus period. Neural activity during stimulus was quantified using the first 60 s of the stimulus. Post- stimulus activity was analyzed over 15 min and subdivided into three consecutive 5- min epochs (0–5, 5–10, 10–15 min).

#### 2.4.3 Novelty suppressed feeding photometry analysis:

In NSF, recordings were obtained during baseline and during exploration/feeding epochs. Behavioral events were manually annotated and timestamped, including food approaches and the first bite.

Event-locked normalization was computed using a local pre- event baseline (3 s pre- event; 5 s post- event window):


$$\frac{\Delta \,F_{465,e\,v\,e\,n\,t}\,\left(t\right)}{{\hat{\bar{F}}}_{465,e\,v\,e\,n\,t}\,\left(t\right)}=\frac{\left(F_{465}\,\left(t\right)-{\hat{\bar{F}}}_{465,e\,v\,e\,n\,t}\right)}{{\hat{\bar{F}}}_{465,e\,v\,e\,n\,t}}$$

Where ${\hat{\bar{F}}}_{465,e\,v\,e\,n\,t}$ is the mean regression-fitted fluorescence during the pre-event window. This approach isolates transient neural activity associated with approach and consummatory behaviors while controlling for slow drift and non-stationary baseline fluctuations. Event-aligned traces were interpolated onto a common time grid and averaged across events within each animal prior to aggregation across animals.

### 2.5 Optogenetic implants and stimulation paradigms

Corticotropin-releasing factor Cre mice received BNST injections of rAAV-Ef1α-flex-hChR2(H134R)-EYFP or rAAV-Ef1α-flex-eGFP. Three optogenetic configurations were used:

1. a. BNST CRF cell bodies: fibers above BNST.
  b. BNST CRF terminals in PVH: fibers above PVH (AP −0.8 mm, ML ± 0.25 mm, DV −4.8 mm).
  c. BNST CRF terminals in LH: fibers above LH (AP −1.3 mm, ML ± 0.93 mm, DV −5.2 mm).

Fiber implants were 200 μm core, 0.22 NA. Optical stimulation used 473 nm illumination at 1 mW at the fiber tip, delivered as 10 Hz pulse trains with 5 ms pulses for 3 min immediately prior to behavioral testing. After experiments, viral expression and fiber placements were histologically verified; animals were excluded for mistargeting or off-target spread (e.g., into hypothalamus or nucleus accumbens).

### 2.6 Indirect calorimetry measurements

Metabolic assessments were performed using CLAMS HC (Oxymax, Columbus Instruments) in the Baylor College of Medicine Mouse Metabolism and Phenotyping Core. Mice were housed individually in metabolic chambers with free access to food and water under a 12 h light/12 h dark cycle (lights on at 06:00, off at 18:00) at 22 °C ± 1 °C. Animals were acclimated for at least 24 h, followed by 96 h of continuous recording.

VO_2_ and VCO_2_ were measured by open circuit calorimetry, energy expenditure (heat production) was calculated using the modified Weir equation, and RER was computed as VCO_2_/VO_2_. Locomotor activity was recorded using infrared beam breaks (X–Y ambulatory and Z rearing). Food and water intake were measured continuously using weighing sensors. Signals were typically aggregated into 1 h bins, with visualization separated by light versus dark phases. Body weight and body composition were measured immediately before and after calorimetry; lean and fat mass were measured by NMR (EchoMRI 100).

All CLAMS experiments were performed with careful attention to animal welfare. The use of pelleted cellulose bedding and familiar food/water sources in the chambers was intended to improve animal comfort and replicate standard housing conditions, thereby reducing stress artifacts. Mice were monitored daily during the study for any signs of stress or irregular behavior.

All components of the indirect calorimetry system (gas analyzers, airflow controllers, and sensors for activity/feeding) were maintained and calibrated according to the manufacturer’s instructions and the consensus guidelines for metabolic phenotyping experiments, ensuring accurate and reproducible measurements of mouse energy homeostasis.

### 2.7 Brain slice electrophysiology

For brain slice preparation, animals were anesthetized with isoflurane and transcardially perfused with ice-cold artificial cerebrospinal fluid (ACSF; pH 7.35, 310 mOsm/L) containing the following (in mM): 125 NaCl, 2.5 KCl, 1.25 NaH_2_PO_4_, 2 CaCl_2_, 1 MgCl_2_, 10 glucose, and 25 NaHCO_3_. Extracted brains were transferred to a sucrose-based dissection solution containing (in mM): 87 NaCl, 2.5 KCl, 1.25 NaH_2_PO_4_, 0.5 CaCl_2_, 7 MgCl_2_⋅6H_2_O, 13 ascorbic acid, 75 sucrose, 10 glucose, and 25 NaHCO_3_, equilibrated with 95% O_2_–5% CO_2_. Coronal forebrain slices (300 μm) containing the BNST were prepared using a Leica VT1200 vibratome and incubated for recovery at 34 °C in oxygenated ACSF for at least 15 min, then gradually equilibrated to room temperature (25 °C) for an additional 15 min prior to recording. The recording chamber was continuously perfused with ACSF at 1–2 mL/min at 25 °C.

Bed nucleus of the stria terminalis CRF neurons were identified by transmitted light differential interference contrast (DIC) microscopy (BX50WI, Olympus) and EGFP fluorescence using 470 nm illumination (Thorlabs DC4100) with 49002-ET-EGFP emission filters (Chroma Technology) and an optiMOS camera (QImaging) controlled by Micro-Manager v1.4.22 software. Whole-cell patch-clamp recordings were performed using an integrated Patch Pro 6000 rig (Scientifica, United Kingdom) with an Axon MultiClamp 700B amplifier, digitized at 10 kHz (Axon Digidata 1440A). Recording electrodes (3–5 MΩ) were fabricated from borosilicate glass microcapillaries (outer diameter: 1.5 mm) using a micropipette puller (Sutter Instruments). The internal solution contained (in mM): 110 K-gluconate, 10 KCl, 4 Mg-ATP, 0.5 Na-GTP, 10 di-Na-phosphocreatine, 1 EGTA, and 10 HEPES; pH adjusted to 7.3 with KOH and osmolarity to 305 mOsm/L with K-gluconate.

Following gigaohm seal formation, baseline firing activity was monitored in cell-attached mode for 5–10 min, then in whole-cell current-clamp configuration for an additional 5–10 min. Hyperpolarizing and depolarizing current injection steps and ramps were applied to characterize intrinsic firing properties, including spontaneous firing pattern, action potential threshold, resting membrane potential, and action potential morphology.

### 2.8 Experimental design and statistical analysis

All experiments were performed using adult male and female CRF- Cre mice. Both sexes were included across experiments; data were pooled when no sex- dependent effects were observed. Animals were assigned to experimental groups based on viral expression (e.g., ChR2 vs. EYFP; NaCh vs. EYFP). Unless otherwise stated, the individual mouse was treated as the experimental unit, and exact sample sizes (n) for each experiment are reported in the corresponding figure legends.

#### 2.8.1 Fiber photometry experiments

For fiber photometry experiments, the experimental unit was the individual mouse. BNST CRF neuronal activity was recorded during exposure to discrete stressors (forced swim stress or predator odor) or during novelty- suppressed feeding. Photometry analyses were structured as within- subject comparisons nested within between- subject analyses. For stressor paradigms, neural activity during stimulus and post- stimulus epochs was quantified relative to a baseline period within each mouse using mean regression-corrected *ΔF/F*. Each mouse contributed a single baseline- normalized value per analysis window, and these values were aggregated across mice for statistical inference.

#### 2.8.2 Optogenetic and behavioral experiments

For optogenetic and chronic behavioral experiments, the experimental unit was the individual mouse. Between- subject factors included viral condition (e.g., ChR2 vs. EYFP; NaCh vs. EYFP) and, where applicable, projection target (BNST→LH vs. BNST→PVH). Behavioral outcomes included time spent in the center of the open field, feeding latency in the novelty- suppressed feeding task, and food intake during fast- refeeding assays. Locomotor activity was analyzed in parallel to assess non-specific motor effects. Pre- stimulation paradigms were used to determine whether transient activation of BNST CRF neurons or their projections was sufficient to bias subsequent behavior.

#### 2.8.3 Indirect calorimetry experiments

For indirect calorimetry experiments, the experimental unit was the individual mouse housed in a metabolic chamber. Prior to experimental manipulation, baseline body weight, food intake, and body composition were measured, and mice were assigned to matched cohorts with maximally similar baseline values across these parameters before random assignment to control or experimental conditions. Energy intake, energy expenditure, respiratory exchange ratio, and locomotor activity were continuously measured and summarized on a per- mouse basis across stable recording periods (e.g., light and dark phases) prior to group- level comparisons.

To guide statistical modeling, relationships between body mass and metabolic variables were first assessed using simple linear regression analyses, pooled across all animals, regardless of treatment group. Body mass was not significantly associated with food intake; therefore, food intake was compared between groups using a between- subject analysis of per- mouse mean values. In contrast, body mass significantly predicted energy expenditure in this preliminary analysis. Accordingly, group differences in energy expenditure were analyzed using analysis of covariance (ANCOVA), implemented as a multiple linear regression model with viral condition as a categorical predictor and body mass as a continuous covariate. Body mass across the recording period was analyzed separately using two- way repeated- measures ANOVA with time and viral condition as factors.

To further assess the reliability of energy expenditure estimates obtained by indirect calorimetry, we examined the relationship between energy balance and change in adipose tissue mass. Because energy intake is measured directly from food consumption and the known caloric density of the diet, concordance between energy balance and adipose tissue change provides an internal validation of the derived energy expenditure values. Pearson correlation and simple linear regression analyses were performed between these variables. Residuals from the linear regression model were z- scored, and values exceeding ± 2 standard deviations were flagged as potential outliers. One data point met this criterion; however, because its inclusion did not alter the interpretation of any findings, it was retained in all analyses to preserve transparency.

A scatter plot with residuals is shown in Supplementary Extended Data Figure 3-1. Supplementary Extended Data Table 1 reports the correlation, regression, and multiple linear regression statistics relating energy balance, adipose tissue change, and energy expenditure, and additionally summarizes the analyses for Figures 3I, J performed without the identified data point.

#### 2.8.4 Brain slice electrophysiology experiments

For brain slice electrophysiology experiments, the experimental unit was the individual neuron. Recordings were obtained from BNST CRF neurons in acute forebrain slices prepared from NaCh-expressing and control CRF-Cre mice. Intrinsic membrane properties — including resting membrane potential, action potential threshold, and spontaneous firing pattern — were compared between groups. Spontaneous firing pattern classifications (silent, single AP, or burst firing) were compared between groups as categorical proportions. Subgroup analyses were additionally performed to characterize membrane properties within firing pattern subgroups (burst-firing and silent cells vs. single AP-firing cells) relative to controls.

### 2.9 Statistical procedures

Statistical analyses were performed using GraphPad Prism. Normality was assessed within each group using Shapiro–Wilk tests. For comparisons against baseline or zero, two- tailed one- sample *t*- tests were used. Between- group comparisons were performed using two- tailed Welch’s unpaired *t*- tests when variances were unequal or Mann–Whitney U tests when data were non- normally distributed. For repeated- measures designs, two- way repeated- measures ANOVA was used where appropriate. For me measures in which body mass significantly predicted the dependent variable (e.g., energy expenditure), group differences were analyzed using analysis of covariance (ANCOVA), implemented as multiple linear regression with experimental condition as a categorical predictor and body mass as a covariate.

All statistical tests were two- tailed with a significance threshold of α = 0.05. Multiple comparisons were controlled through predefined analysis windows and planned comparisons; *post hoc* exploratory analyses were performed only when explicitly stated. Complete statistical results for all figures and panels are reported in Supplementary Extended Data Table 1.

## 3 Results

### 3.1 BNST CRF neurons are recruited by acute stressors that promote avoidance and food-related approach/avoidance conflict

Stressors may be defined as stimuli that threaten survival, eliciting a broad range of context-appropriate behavioral adaptations that prioritize protection of the organism (Fanselow, 1994; Hoffman et al., 2022; Holly et al., 2016). A hallmark of this response is avoidance and suppression of competing needs, such as feeding (Rosen and Schulkin, 1998; Endres et al., 2005). This trade-off is well-illustrated by the Novelty-Suppressed Feeding Test (NSFT), where, despite significant hunger, fasted mice placed in a novel environment delay approaching and consuming food (Samuels and Hen, 2011). Similarly, predator odor — an ethologically relevant threat cue — evokes robust avoidance and transient hypophagia (Villalobos et al., 2023; Herman and Valone, 2000). Prior work further demonstrates that forced swim, an inescapable physical stressor, similarly drives avoidance-oriented behaviors (Kudryashov et al., 2025) and acutely suppresses feeding during and immediately following stress exposure (de Araujo Salgado et al., 2023). To verify that observed ΔF/F signals reflected calcium-dependent neural activity rather than motion artifacts, 405 nm isosbestic control traces were examined across all animals and sessions. For stressor paradigms, isosbestic signals remained stable within each recording epoch, with expected motion-related transients at stressor onset that were corrected by the regression-based normalization procedure. For NSFT recordings, event-locked 405 nm traces showed no systematic deflection at approach or consumption events, confirming that the opposing ΔF/F responses observed in the 465 nm channel reflect genuine calcium-dependent activity (Supplementary Extended Data Figure 1-1).

**FIGURE 1:**
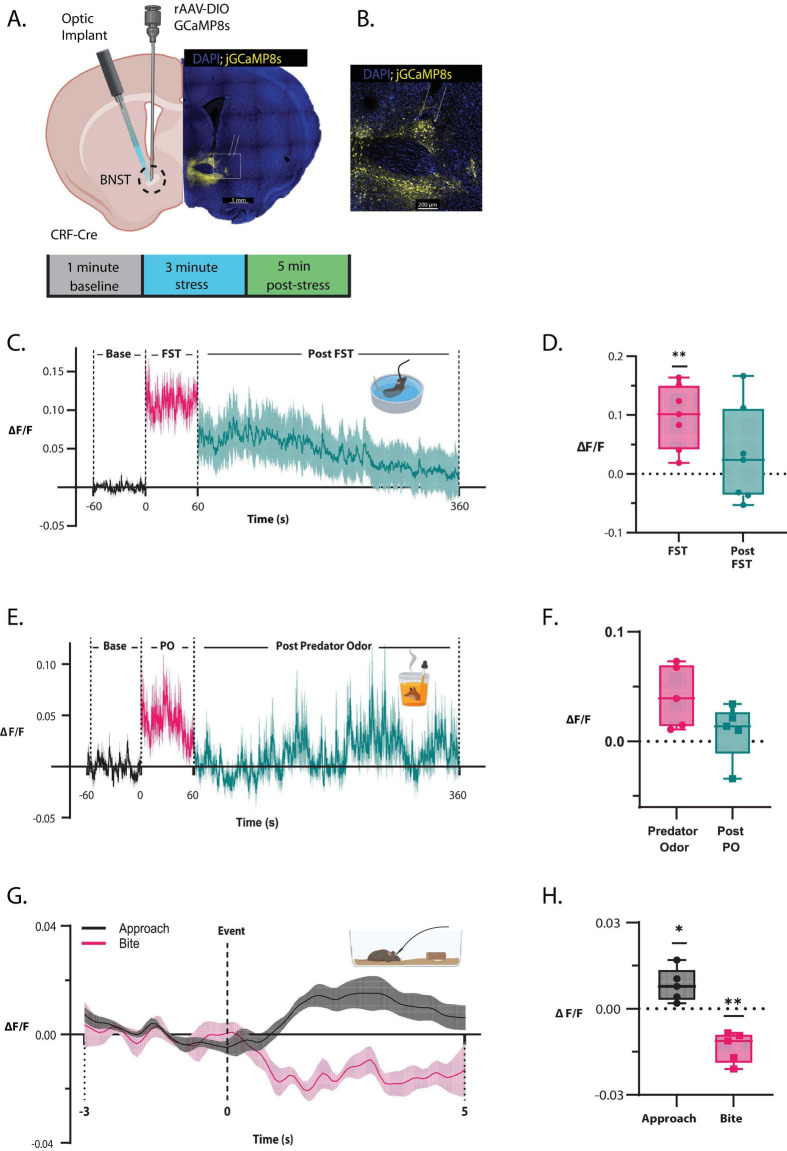
Bed nucleus of the stria terminalis (BNST) corticotropin-releasing factor (CRF) neurons are activated by stressful stimuli and show state-dependent activity during feeding in a conflict-based assay. **(A)** Experimental timeline and schematic of fiber photometry recordings from BNST CRF neurons. CRF-Cre mice received unilateral injection of Cre-dependent jGCaMP8s into BNST and implantation of an optical fiber above BNST. Representative coronal hemisection shows jGCaMP8s expression and fiber placement. **(B)** Higher magnification of region outlined in panel **(A)**. **(C)** Mean **Δ**F/F trace during baseline (black), forced swim stress (pink), and post-stress period (cyan). **(D)** Quantification of mean **Δ**F/F during forced swim and post-stress epochs relative to baseline (baseline set to 0). **(E)** Mean **Δ**F/F trace during baseline (black), predator odor exposure (pink), and post-odor period (cyan). **(F)** Quantification of mean **Δ**F/F during predator odor and post-odor epochs relative to baseline (baseline set to 0). **(G)** Mean **Δ**F/F trace during novelty-suppressed feeding (NSF) aligned to behavioral events (t = 0 denotes approach or consumption onset as indicated). **(H)** Event-locked quantification of mean **Δ**F/F during approach and consumption epochs relative to the 3 s pre-event baseline (quantified over the 5 s post-event window). Baseline for stressor paradigms was defined as −70 to −10 s relative to stressor onset; stressor epochs were 180 s, but only 60 s are visualized; post-stressor epochs were 15 min, but only the first 5 min are visualized. All traces reflect 2 Hz low-pass filtered **Δ**F/F signals, consistent with the signals used for all statistical analyses. No additional smoothing was applied. Traces are presented as mean ± SEM. Box-and-whisker plots show median (center line), interquartile range (box), and min–max (whiskers). Statistical significance in panels **(D,F,H)** was assessed using two-tailed one-sample *t*-tests against 0 (baseline-normalized **Δ**F/F). **(D)** Forced swim: FST vs. 0, ***p*** = 0.0031; post-FST vs. 0, *p* = 0.3596. **(F)** Predator odor: odor vs. 0, *p* = 0.0385; post-odor vs. 0, *p* = 0.7306. **(H)** NSF event-locked: novel approach vs. 0, *p* = 0.0326; novel consumption vs. 0, *p* = 0.0177; home-cage approach vs. 0, *p* = 0.4777; home-cage consumption vs. 0, *p* = 0.3379. Sample sizes: **(C,D)**, forced swim photometry *n* = 7 mice; **(E,F)** predator odor photometry *n* = 5 mice; **(G,H)** NSF photometry *n* = 5 mice. Each point represents one mouse. Male and female mice were pooled. Significance: *p < 0.05, **p < 0.01. Created with BioRender.

Observations that stressors drive avoidance raised the question of whether these qualitatively distinct stressors may converge on shared neural circuits. The BNST is well-positioned to mediate such convergence, and its CRF-expressing neurons in particular are known to relay stress and threat information (Dabrowska et al., 2013). Indeed, previous studies have shown increased c-Fos expression in the BNST following avoidance-driving stimuli (Butler et al., 2016). However, the temporal dynamics of BNST CRF recruitment, and whether it generalizes across qualitatively distinct stressor categories, remain unresolved. To address this, we first performed fiber photometry calcium recordings by targeting expression of Cre-dependent jGCaMP8s (Zhang et al., 2023) to the BNST of CRF-Cre mice (Taniguchi et al., 2011). Following viral injections, fiber optic implants, and 5 weeks of recovery, we recorded calcium dynamics during exposure to three qualitatively distinct stressors: fox urine (predator odor), forced swim (inescapable physical stress), and novelty-suppressed feeding (Figures 1A, B).

Interestingly, forced swim drove robust, sustained increases in BNST CRF neuron activity [Figures 1C, D; one sample *t*-test, *p* = 0.0031, *t*(6) = 4.762, r^2^ = 0.791]. However, upon removal of the stressor, BNST CRF dynamics returned to baseline [Figure 1C; one sample *t*-test, *p* = 0.360, *t*(6) = 0.992, r^2^ = 0.141]. Similarly, exposure to fox urine led to increased BNST CRF calcium dynamics [Figures 1E, F; one sample *t*-test: *p* = 0.039, *t*(4) = 3.037, r^2^ = 0.700]. However, activity returned to baseline following removal of the stressor [Figure 1F; one sample *t*-test: *p* = 0.731, *t*(4) = 0.369, r^2^ = 0.033]. We next examined BNST CRF dynamics during the approach–avoidance conflict inherent to the NSFT. For this we first placed fasted mice in a novel, brightly lit arena with a food pellet at the center. While the mouse explored the arena, we tracked BNST CRF activity as mice moved toward the pellet (without eating) and during actual food consumption. We noted that BNST CRF activity rose markedly during tentative approach in the novel arena when faced with the drive to eat and the urge to remain cautious [Figures 1G, H; one sample *t*-test: *p* = 0.0326, *t*(4) = 3.21, r^2^ = 0.721]. In contrast, approaches that resulted in feeding were correlated with a significant reduction in BNST CRF activity [Figures 1G, H; one sample *t*-test: *p* = 0.0177, *t*(4) = 3.886, r^2^ = 0.791].

Collectively, these data demonstrate that BNST CRF neurons respond to qualitatively distinct stressors, including physical (forced swim), olfactory (predator odor), and contextual (novel environment), and that their activities are highest during times of conflict between avoidance and competing drives such as hunger. These findings position BNST CRF neurons as a convergence point for diverse threat signals.

### 3.2 Acute and chronic activation of BNST CRF neurons drives avoidance behaviors

Our photometry data demonstrated that BNST CRF neurons were robustly recruited by qualitatively distinct stressors. Prior work has further implicated CRF-expressing BNST neurons in driving avoidance-related behaviors (Zhang et al., 2024; Giardino et al., 2018; Hu et al., 2020; Baumgartner et al., 2021). However, whether selective recruitment of this population is sufficient to bias subsequent behavior toward avoidance remains unknown. We therefore asked whether targeted activation of BNST CRF neurons alone is sufficient to promote avoidance behavior.

**FIGURE 2:**
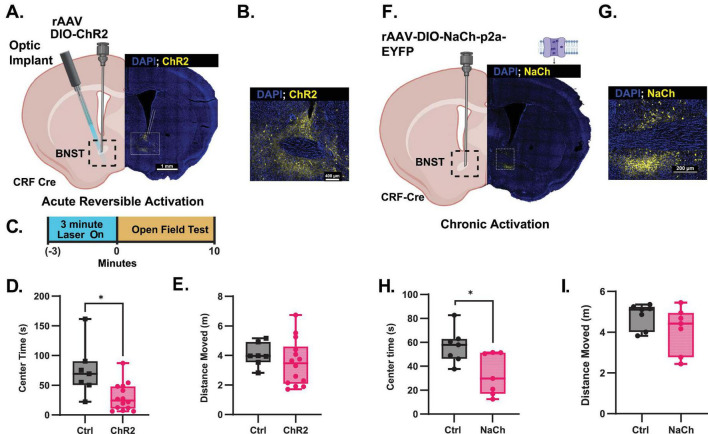
Acute and chronic activation of bed nucleus of the stria terminalis (BNST) corticotropin-releasing factor (CRF) neurons increase avoidance-like behavior. **(A)** Strategy for acute optogenetic activation of BNST CRF neurons. CRF-Cre mice received bilateral BNST injections of Cre-dependent ChR2 or control EYFP and bilateral fibers above BNST. **(B)** Representative coronal section showing BNST ChR2 expression and fiber placement. **(C)** Pre-stimulation paradigm (3 min stimulation delivered in the home cage immediately prior to open field testing; no stimulation during the assay). **(D)** Time spent in the center of the open field. **(E)** Distance moved during the open field. **(F)** Strategy for chronic activation of BNST CRF neurons (NaCh expression) in CRF-Cre mice. **(G)** Representative expression inset. **(H)** Time spent in the center of the open field following chronic activation. **(I)** Distance moved following chronic activation. Box-and-whisker plots show median (center line), interquartile range (box), and min–max (whiskers). Statistical significance was assessed using two-tailed Welch’s unpaired *t*-tests. **(D)** Acute pre-stimulation: center time *p* = 0.0358; **(E)** distance moved *p* = 0.3087. Chronic NaCh: **(H)**, center time *p* = 0.0190; (I) distance moved *p* = 0.1987. Sample sizes: **(D,E)** acute ChR2 pre-stimulation ChR2 *n* = 14 mice, EYFP *n* = 7 mice; chronic NaCh **(H,I)**, *n* = 7 mice/group. Male and female mice were pooled. Significance: *p < 0.05. Created with BioRender.

To selectively target BNST CRF neurons for activation, we virally expressed Cre-dependent Channelrhodopsin-EYFP (ChR2) or EYFP control virus in the BNST of CRF-Cre mice (Figures 2A, B). Following recovery, mice underwent open field testing (OFT) to assess avoidance behavior. To parallel the transient recruitment of BNST CRF neurons observed during stress exposure in our photometry experiments, we delivered 3 min of optogenetic stimulation (10 Hz, 5 ms pulses) prior to OFT (Figure 2C). This design allowed us to test whether a brief episode of BNST CRF neuron activation was sufficient to influence immediately subsequent behavioral responses in anxiogenic environments.

Indeed, pre-stimulation of BNST CRF neurons significantly reduced the amount of time ChR2-expressing mice spent in the center of the open field relative to EYFP controls, indicating increased avoidance behavior [Figure 2D; Welch’s *t*-test: *p* = 0.0358, *t*(7.756) = 2.536, r^2^ = 0.4532]. Notably, locomotor activity was unchanged between groups, suggesting that reduced center exploration did not result from altered ambulatory drive [Figure 2E; Welch’s *t*-test: *p* = 0.309, *t*(18.94) = 1.046, r^2^ = 0.055]. Together, these findings demonstrate that transient pre-stimulation of BNST CRF neurons was sufficient to bias animals toward avoidance behaviors. The temporal persistence of this effect beyond stimulation offset was not characterized.

We next asked whether sustained elevation of BNST CRF neuron output would similarly promote avoidance. Toward this, we expressed a Cre-dependent sodium bacterial channel (NaCh) in BNST CRF neurons (Figures 2F, G). NaCh lowers the threshold for action potential generation and enhances neuronal output (Sim et al., 2013; Patel et al., 2019). To validate functional NaCh expression in BNST CRF neurons specifically, we performed whole-cell patch-clamp recordings in acute brain slices, confirming significantly more negative AP threshold in NaCh-expressing neurons compared to controls [Supplementary Extended Data Figure 2-1: Welch’s *t*-test: *p* < 0.0067, *t*(17.6) = 3.078, r^2^ = 0.354], with characteristically wide action potentials observed in all NaCh-expressing cells upon current injection.

Two weeks following viral delivery, NaCh-expressing mice likewise spent significantly less time in the center of the open field compared to controls [Figure 2H; Welch’s *t*-test: *p* = 0.019, *t*(11.62) = 2.722, r^2^ = 0.380], without changes in locomotion [Figure 2I; Welch’s *t*-test: *p* = 0.199, *t*(9.410) = 1.383, r^2^ = 0.169]. In sum, these data establish that both transient and sustained activity manipulations of BNST CRF neuron activity are sufficient to promote avoidance behavior.

### 3.3 Activation of BNST CRF neurons suppresses feeding

**FIGURE 3:**
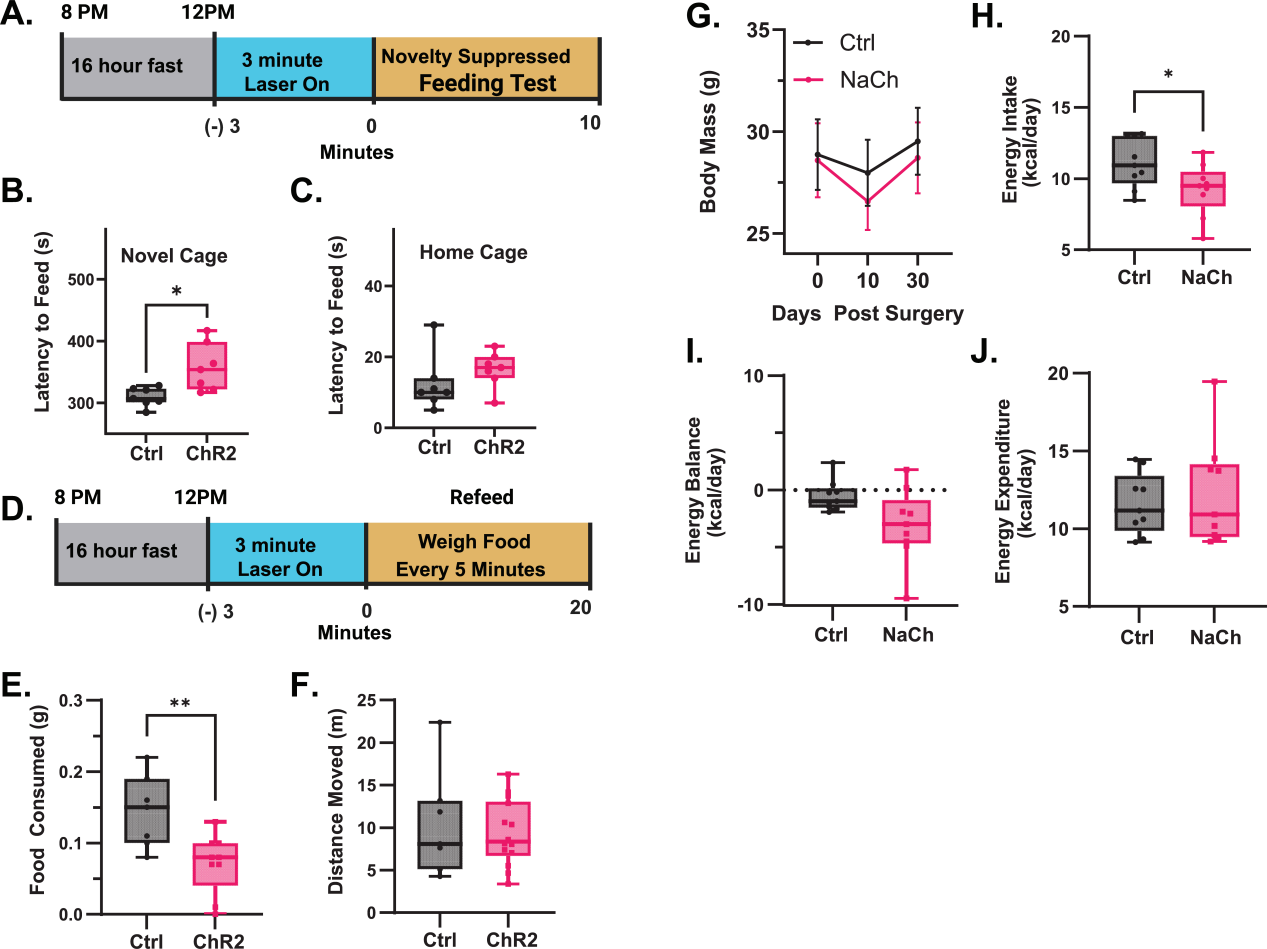
Acute and chronic activation of bed nucleus of the stria terminalis (BNST) corticotropin-releasing factor (CRF) neurons differentially affect feeding. **(A)** Pre-stimulation paradigm used during novelty-suppressed feeding (NSF) prior to testing. **(B)** Latency to initiate feeding in the novel arena during NSF. **(C)** Latency to initiate feeding in the home cage. **(D)** Pre-stimulation paradigm used for the fast-refeeding assay. **(E)** Food consumed during the refeeding period. **(F)** Locomotor activity during refeeding. **(G)** Body mass over time during indirect calorimetry recordings in NaCh and control mice, including measurements at baseline, day 10, and day 30 post-viral delivery. Measurements of **(H)** Energy Intake, **(I)** energy balance, and **(J)** energy expenditure while mice were within indirect calorimetry cages. Box-and-whisker plots show the median (center line), interquartile range (box), and minimum–maximum values (whiskers); each point represents one mouse. **(B–F)** Acute optogenetic assays were analyzed using two-tailed Welch’s unpaired *t*-tests: **(B)** NSF novel latency *p* = 0.0116; **(C)** NSF home latency, *p* = 0.5763; **(E)** fast-refeeding intake, *p* = 0.0205; **(F)** refeeding locomotion, *p* = 0.9581. **(G)** Body mass over time was analyzed using two-way repeated-measures ANOVA (time × viral condition interaction, *p* = 0.543; main effect of time, *p* = 0.0032; main effect of viral condition, *p* = 0.719); *post hoc* Sidak’s comparisons revealed a transient reduction in body mass at day 10 in NaCh-expressing mice [*p* = 0.024, *t*(32) = 2.83, r^2^ = 0.200] that resolved by day 30 [*p* = 0.998, *t*(32) = 0.172, r^2^ = 0.001]. CLAMS summary measures were analyzed using either two-tailed Welch’s unpaired *t*-tests: **(H)** energy intake, *p* = 0.0420; **(I)** energy balance, *p* = 0.0515 or ANCOVA: implemented as multiple linear regression with body mass as a covariate; **(J)** energy expenditure, overall model *p* = 0.163, viral treatment *p* = 0.782. Sample sizes: **(B–F)** acute optogenetic NSF and feeding assays, control *n* = 7 mice, ChR2 *n* = 14 mice; **(G–J)** chronic NaCh indirect calorimetry, *n* = 9 mice per group. Male and female mice were pooled. Significance: *p < 0.05, **p < 0.01. Created with BioRender.

Having established that both acute and sustained manipulations of BNST CRF neurons were sufficient to promote avoidance behavior, and that these neurons are differentially engaged during the novelty suppressed feeding test, with increased activity during approach without bite and decreased activity during approach with bite, we next asked whether engaging this population also influences feeding behavior. This question was motivated by the close association between avoidance behaviors and eating disorders in humans (Swinbourne et al., 2012; Ngan et al., 2017; Eck and Byrd-Bredbenner, 2021; Godart et al., 2000), as well as evidence that the BNST contributes to the regulation of energy balance during stress (Wang et al., 2025, 2019; Douglass et al., 2023). To next test whether acute BNST CRF neuron activation influenced feeding behavior in a conflict-based context, we performed the novelty suppressed feeding test (NSFT) in mice expressing ChR2 or EYFP control virus. In this assay, food-deprived animals must decide whether to initiate feeding in a novel, anxiogenic environment, measuring latency to feeding initiation with competing approach and avoidance demands. For this, we implanted fiber optics over the BNST and delivered a brief three-minute period of photostimulation (10 Hz, 5 ms pulse duration) immediately prior to NSFT (Figure 3A). In the novel environment, ChR2-stimulated mice exhibited a significantly increased latency to initiate feeding relative to EYFP controls [Figure 3B; Welch’s *t*-test: *p* = 0.0116, *t*(15.93) = 2.849, r^2^ = 0.338], indicating that prior BNST CRF neuron activation biased against feeding under conditions of environmental challenge. In contrast, no difference in feeding latency was observed when the same animals were tested in the home cage [Figure 3C; Welch’s *t*-test, *p* = 0.576, *t*(10.48) = 0.577, r^2^ = 0.031], suggesting that prior BNST CRF neuron activation does not broadly impair the initiation of feeding when competing environmental demands are minimal. It should be noted that within the NSF arena, increased thigmotaxis and feeding latency are spatially related, as animals remaining at the periphery are physically farther from the centrally placed food; the latency measure therefore reflects a combination of avoidance and feeding suppression that cannot be fully dissociated within this paradigm alone.

Because the NSFT latency measure primarily reflects the decision to initiate a feeding bout, we next asked whether BNST CRF neuron activation also influences gross consummatory feeding during the refeeding period in a familiar environment. To ensure familiarity, mice were first habituated to the testing environment for 5 days, for 20 min at a time. We used a fast-refeeding assay in which mice were food-deprived overnight and then subjected to 3 min of photostimulation immediately prior to refeeding, with no stimulation delivered during the refeeding period itself (Figure 3D). Under these conditions, ChR2-expressing mice consumed significantly less food during the refeeding period compared to EYFP controls [Figure 3E; Welch’s *t*-test: *p* = 0.021, *t*(10.35) = 2.731, r^2^ = 0.419]. Locomotor activity during refeeding did not differ between groups [Figure 3F; Welch’s *t*-test: *p* = 0.958, *t*(10.87) = 0.0537, r^2^ = 0.000266], indicating that reduced food intake was not attributable to altered motor output. Together, these data show that acute activation of BNST CRF neurons is sufficient to reduce consummatory food intake in a fast-refeeding paradigm.

Together, these findings indicate that BNST CRF neuron activation does not uniformly suppress feeding, but instead differentially influences distinct components of feeding behavior. Specifically, BNST CRF activation biases away from the initiation of feeding under environmental challenge, while also reducing total food consumption during refeeding. This dissociation suggests that BNST CRF circuitry can influence multiple dimensions of feeding behavior depending on task demands, rather than exerting a single, context-independent effect on feeding.

Given the observed acute effects on feeding initiation and consumption, we next asked whether sustained BNST CRF neuron activation produces longer-lasting alterations in feeding behavior. To address this, we expressed Cre-dependent NaCh or EYFP control virus in the BNST of CRF-Cre mice and assessed energy intake and expenditure using comprehensive laboratory animal monitoring systems (CLAMS) following recovery and habituation.

Across the recording period, NaCh-expressing and control mice exhibited comparable body mass, with no significant main effect of viral treatment and no interaction between viral treatment and time. However, in the NaCh group, body mass varied across the recording period, with a transient reduction at day 10 relative to baseline [Sidak’s multiple comparisons: *p* = 0.0239, *t*(32) = 2.827, r^2^ = 0.200] that resolved by day 30 [Sidak’s multiple comparisons: *p* > 0.9975, *t*(32) = 0.172, r^2^ = 0.001]. No significant differences were observed in the control group [Figure 3G; two- way RM ANOVA: viral treatment, *F*(1,16) = 0.1334, *p* = 0.720, η^2^p = 0.008; time × viral treatment, *F*(2,32) = 0.622, *p* = 0.543, η^2^p = 0.037; time, *F*(2,32) = 7.41, *p* = 0.002, η^2^p = 0.316].

Because body mass is generally assumed to influence both food intake and energy expenditure, we explicitly tested this assumption prior to group comparisons. Simple linear regression revealed no significant relationship between body mass and energy intake (r^2^ = 0.146, *p* = 0.118), indicating that variability in feeding was not attributable to differences in body weight. Accordingly, between- group comparisons showed that NaCh- expressing mice exhibited reduced energy intake relative to controls [Figure 3H; Welch’s *t*- test: *t*(15.95) = 2.211, *p* = 0.0420, r^2^ = 0.235]. Despite this reduction in intake, overall energy balance did not differ significantly between groups by conventional thresholds [Figure 3I; Welch’s *t*- test: *t*(10.70) = 2.191, *p* = 0.0515, r^2^ = 0.31, CI: −5.125 to 0.020]. However, the large effect size and confidence interval approaching zero are consistent with a trend toward negative energy balance that is concordant with the transient body weight reduction observed at day 10.

In contrast, simple linear regression confirmed a significant positive association between body mass and energy expenditure across animals (r^2^ = 0.425, *p* = 0.0034), motivating its inclusion as a covariate in subsequent group comparisons. Therefore, energy expenditure was analyzed using multiple linear regression (ANCOVA) including body mass, viral treatment, and their interaction. In this model, neither body mass [*F*(1,14) = 1.919, *p* = 0.1877], viral treatment [*F*(1,14) = 0.0795, *p* = 0.7821], nor their interaction [*F*(1,14) = 0.1430, *p* = 0.7110] significantly predicted energy expenditure, and the overall model was not significant [Figure 3J; *F*(3,14) = 1.986, *p* = 0.1625]. The non-significant body mass term in this model reflects the expected attenuation of its partial effect once viral treatment and their interaction are included as additional predictors, and does not contradict the significant association observed in the preliminary pooled regression.

Together, these findings indicate that sustained BNST CRF neuron activation selectively suppresses food intake, trends toward decreased energy balance, and transiently reduces body mass, without significantly altering energy expenditure. These data demonstrate that BNST CRF circuitry contextually biases feeding behavior while leaving systemic energy homeostasis largely intact.

### 3.4 BNST CRF neurons project to hypothalamic circuits that drive both avoidance and feeding suppression

**FIGURE 4:**
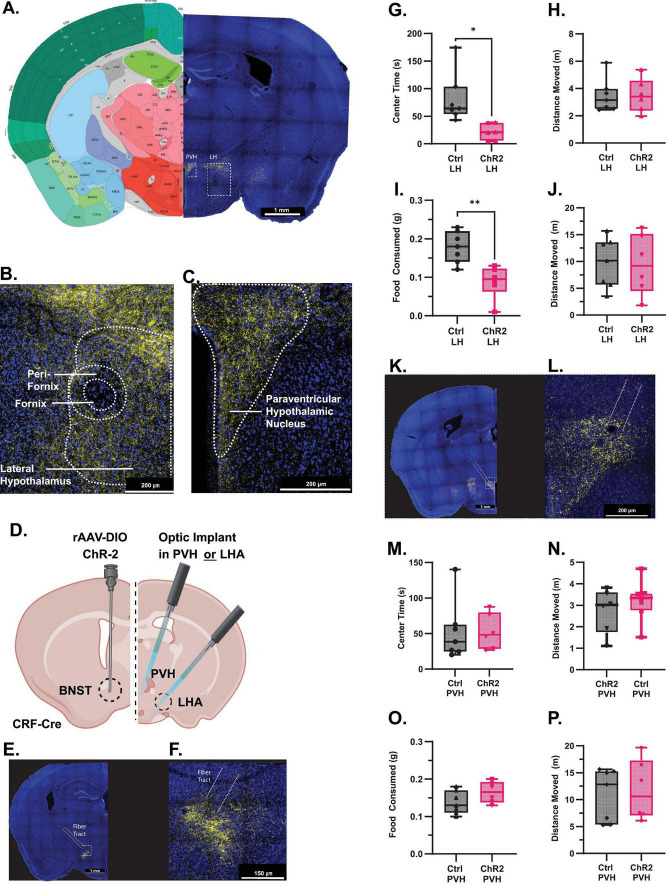
Bed nucleus of the stria terminalis (BNST) corticotropin-releasing factor (CRF) projections to hypothalamic targets differentially regulate avoidance behavior and feeding. **(A)** Allen Brain Institute Reference atlas hemisection (Interactive Atlas Viewer:Atlas Viewer) illustrating regions enlarged in panels **(B,C)** (PVH and LH indicated). **(B)** Higher magnification view of LH terminal labeling. **(C)** Higher magnification view of PVH terminal labeling. **(D)** Terminal field optogenetic strategy: CRF-Cre mice received BNST injections of Cre-dependent ChR2 or control EYFP and fibers above LH or PVH. **(E)** Representative hemisection showing BNST CRF axon labeling in LH and fiber placement. **(F)** Inset of boxed region in panel **(E)**. (G) Open field center time following BNST→LH terminal pre-stimulation. **(H)** Open field distance moved following BNST→LH terminal pre-stimulation. **(I)** Food consumed during fast-refeeding following BNST→LH terminal pre-stimulation. (J) Locomotor activity during refeeding following BNST→LH terminal pre-stimulation. (K) Representative hemisection showing BNST CRF axon labeling in PVH and fiber placement. **(L)** Inset of boxed region in panel **(K)**. **(M)** Open field center time following BNST→PVH terminal pre-stimulation. (N) Open field distance moved following BNST→PVH terminal pre-stimulation. **(O)** Food consumed during fast-refeeding following BNST→PVH terminal pre-stimulation. **(P)** Locomotor activity during refeeding following BNST→PVH terminal pre-stimulation. Box-and-whisker plots show median (center line), interquartile range (box), and min–max (whiskers); each point represents one mouse. Separate EYFP control cohorts were used for LH and PVH terminal stimulation experiments. For center-time comparisons where normality was not met in controls, two-tailed Mann–Whitney tests were used: **(G)** BNST→ LH center time **p** = 0.0012; **(M)** BNST→ PVH center time *p* = 0.5338. Other panels were analyzed using two-tailed Welch’s unpaired *t*-tests: **(H)** LH distance moved *p* = 0.9658; **(I)** LH fast-refeeding intake *p* = 0.0028; **(J)** LH refeeding locomotion *p* = 0.7056; **(N)** PVH distance moved *p* = 0.4107; **(O)** PVH fast-refeeding intake *p* = 0.1187; **(P)** PVH refeeding locomotion *p* = 0.9562. Sample sizes: LH cohort—ChR2 *n* = 6 mice, EYFP *n* = 7 mice; PVH cohort—ChR2 *n* = 6 mice, EYFP *n* = 7 mice. Male and female mice were pooled. Significance: *p < 0.05, **p < 0.01. Created with BioRender.

Understanding how a single neuronal population coordinates diverse behavioral outputs requires identifying its downstream targets. The BNST projects broadly to brain regions involved in autonomic regulation, energy homeostasis, rewards, and motivation—including the hypothalamus, brainstem, and nucleus accumbens—positioning it at an anatomical crossroads linking anxiety, motivation, and metabolic regulation (Sun et al., 2023; Hao et al., 2019; Giardino et al., 2018; Kim et al., 2013). Prior anatomical tracing studies, including those catalogued in the Allen Brain Connectivity Atlas (Projection Experiment Page::Allen Brain Atlas: Mouse Connectivity and Projection Experiment Page::Allen Brain Atlas: Mouse Connectivity), have identified several projection targets of BNST CRF neurons, notably the paraventricular hypothalamic nucleus (PVH), lateral hypothalamic nucleus (LH) and amygdala (Oh et al., 2014). Given the established roles of both structures in avoidance and feeding control, we reasoned that projections to the PVH and LH were the strongest candidates for coupling BNST CRF–driven avoidance states to changes in feeding behavior. To first validate that BNST CRF neurons project to the LH and PVH, we employed a cell type specific anterograde labeling strategy. For this, we expressed a Cre dependent rAAV encoding synaptophysin GFP in the BNST of CRF Cre mice (Figure 4A). Expression of synaptophysin:GFP labels axon terminals and putative synaptic targets of BNST CRF neurons (Li et al., 2010). Two weeks after injection, histological analysis revealed robust synaptophysin GFP–positive terminal fields in both the LH (Figure 4B) and PVH (Figure 4C), consistent with previously reported connectivity.

Having confirmed these projection pathways, we next asked whether BNST CRF→LH and BNST CRF→PVH projections contribute to avoidance and feeding behaviors. Whereas our prior experiments activated BNST CRF cell bodies, thereby engaging all downstream targets simultaneously, we next sought to isolate projection specific contributions by selectively activating axon terminals at each site. We reasoned that if pre-stimulation of a discrete BNST CRF projection recapitulates behavioral effects observed with cell body activation, then that projection represents a sufficient downstream node for recapitulating the prior observed behaviors. Still, axon terminal photostimulation may antidromically activate cell bodies, which could in turn engage collateral projections beyond the target site. Thus, any observed phenotypes should be interpreted as evidence of sufficiency rather than strict pathway specificity.

To test this, we stereotaxically delivered Cre dependent ChR2 EYFP or EYFP control virus to the BNST of CRF Cre mice (Figure 4D) and implanted fiber optics over either the LH or PVH. Following recovery, we delivered three minutes of photostimulation (10 Hz, 5 ms pulse duration) immediately prior to open field testing.

We first examined the behavioral consequences of selectively stimulating BNST CRF→LH projections. Representative images confirming LH targeting are shown in Figures 4E, F (global view and inset). Pre stimulation of BNST CRF→LH axon terminals significantly reduced time spent in the center of the open field in ChR2 expressing mice relative to EYFP controls (Figure 4G; Mann-Whitney: U = 0, *p* = 0.0012, *r* = 1.0), indicating that activating only this projection was sufficient to drive avoidance behavior. Notably, locomotion did not differ between groups [Figure 4H; Welch’s *t*-test: *p* = 0.966, *t*(10.66) = 0.0438, r^2^ = 0.00018], confirming that reduced center exploration reflected avoidance rather than altered motor output. We next asked whether the BNST CRF→LH projection also contributes to feeding suppression. Using the same pre stimulation protocol, we performed the fast-refeeding assay following an overnight fast. Pre stimulation of BNST CRF→LH terminals significantly reduced food intake during the refeeding period in ChR2 expressing mice compared to controls [Figure 4I; Welch’s *t*-test: *p* = 0.0028, *t*(10.55) = 3.873, r^2^ = 0.5871], without altering locomotion [Figure 4J; Welch’s *t*-test: *p* = 0.7056, *t*(7.787) = 0.392, r^2^ = 0.0194]. Together, these findings demonstrate that activating the BNST CRF→LH projection was sufficient to drive both avoidance and suppression of food intake—recapitulating the cardinal behavioral outputs observed with BNST CRF cell body activation.

We next examined BNST CRF→PVH projections. Representative images confirming PVH targeting are shown in Figures 4K, L (global view and inset). In contrast, pre-stimulation of BNST CRF→PVH terminals did not significantly alter time spent in the center of the open field in ChR2-expressing mice relative to controls (Figure 4M; Mann-Whitney: U = 16, *p* = 0.5338, *r* = 0.24), locomotion [Figure 4N; Welch’s *t*-test: *p* = 0.411, *t*(10.41) = 0.857, r^2^ = 0.069], food intake during fast-refeeding [Figure 4O; Welch’s *t*-test: *p* = 0.119, *t*(10.86) = 1.694, r^2^ = 0.209], or locomotor activity during refeeding [Figure 4P; Welch’s *t*-test: *p* = 0.956, *t*(7.663) = .0568, r^2^ = 0.000420]. These data indicate that the PVH projection is not sufficient to mediate either avoidance or feeding suppression in this context.

Collectively, these findings identify the BNST CRF→LH pathway as a key downstream circuit through which BNST CRF neurons coordinate avoidance states with suppression of feeding. The inability of PVH terminal stimulation to recapitulate either phenotype indicated that these behavioral effects are not a general property of all BNST CRF projections, but instead arise from anatomically specific downstream pathways. More broadly, these results suggest that BNST CRF neurons orchestrate a multidimensional behavioral repertoire, encompassing avoidance and feeding suppression, as well as gating between the two actions. This occurs through discrete projection pathways, with the LH serving as a critical effector site for coupling threat related behavioral states to the regulation of feeding.

## 4 Discussion

This study identifies a genetically defined population of corticotropin-releasing factor (CRF)–expressing neurons in the bed nucleus of the stria terminalis (BNST) that mediate avoidance-related and feeding behaviors. Using fiber photometry, we demonstrate that diverse stressors evoke time-locked calcium transients in BNST CRF neurons, indicating rapid and dynamic recruitment by threat. Calcium recordings during the novelty-suppressed feeding test correlate BNST CRF suppression and the initiation of feeding in anxiogenic environments. These data suggest that the BNST CRF population is a node through which threat-related signals suppress feeding when hunger and threat compete. Both acute and chronic activation of BNST CRF neurons was sufficient to induce avoidance phenotypes accompanied by coordinated reductions in food intake. Terminal field optogenetic manipulations further implicate the lateral hypothalamus (LH) as a downstream structure sufficient for both feeding suppression and avoidance behaviors.

### 4.1 Stress and the neural correlates of defensive behaviors

To enhance survival, defensively avoidant states are generated in ambiguously threatening contexts, such as acute restraint, foot-shock, and exposure to predator odor (Liu et al., 2023; Xu et al., 2023; Mitten et al., 2024; Nasca et al., 2013; Daviu et al., 2019). Interestingly, behavioral avoidance is strongly associated with several psychiatric illnesses (Hayes et al., 1996; Sporrer et al., 2026), including eating disorders (Collantoni et al., 2024; Frank, 2021; Melles et al., 2021), anxiety disorders (Terrill et al., 2024; Beesdo et al., 2007; Craske et al., 2017; Pittig et al., 2021), affective disorders (Wardenaar et al., 2012; Ironside et al., 2020), and substance-use disorders (Eberl et al., 2013). Thus, understanding the neurobiological substrate underpinning defensive avoidance and its contributions to psychopathology is important. Studies have revealed the involvement of the BNST in stress adaptation (Williford et al., 2023; Mazzone et al., 2018; Sink et al., 2013; Walker et al., 2009; Pedersen et al., 2019). CRF neurons within the BNST are an interesting population that link stress exposure to behavioral changes (Rasiah et al., 2023; Daviu and Bains, 2021; Chang and Deussing, 2021; Hon et al., 2025). We used *in vivo* calcium imaging during avoidance-inducing stressors to show immediate and dynamic recruitment of BNST CRF neuron activity that reverted back to baseline upon removal of the stressor. This mirrors similar activity profiles of the CRF population in the paraventricular hypothalamic nucleus (PVH CRF), which also serve to translate physiologic stress toward physiologic and behavioral adaptation (Füzesi et al., 2016).

### 4.2 Neural substrates driving defensive behaviors

To better understand how BNST CRF activity drives defensive avoidance and feeding phenotypes, we developed an optogenetic pre-stimulation paradigm whereby BNST CRF neurons were stimulated for three minutes prior to either open field test (OFT) or refeed. Previous studies have attempted to unravel the complexity of the cell type-specific BNST-mediated anxiety, and targeted activity manipulations have identified glutamatergic (Jennings et al., 2013b), Pnoc-ergic (Rodriguez-Romaguera et al., 2020), and PKC-δ-expressing (Williford et al., 2023) neurons as drivers of avoidance. While some of these studies have explored calcium dynamics during stress and avoidance, they have not explored how pre-stimulation of a population effects eventual expression of avoidance behaviors. Thus, our findings that show that pre-stimulation of BNST CRF neurons is sufficient to decrease both center time in the OFT and refeed following a fast, are significant because they identify a candidate upstream, state-setting node whose activation during a stressor is sufficient to drive downstream circuit changes that shift animal behavior toward avoidance. How this occurs is a matter of future investigation, however, there are two likely possibilities. Most parsimoniously, the neuromodulatory nature of CRF signaling enables sustained changes to downstream circuits (Hu et al., 2020; Donner et al., 2020; Vandael et al., 2021) that may drive direct behavioral outputs implicated in feeding and center-avoidance. Alternatively, BNST CRF neurons are GABAergic, and thus, may modulate the tone of downstream neuromodulatory centers. For example, image datasets from the Allen Brain Connectivity Atlas show BNST CRF neurons strongly innervate the periaqueductal gray, pallidum and striatum (Oh et al., 2014). These mechanistic possibilities are not mutually exclusive, and distinguishing between them will require future experiments directly manipulating CRF peptide signaling independently of GABAergic transmission during the post-stimulation behavioral window. Regardless of the precise mechanism, the behavioral consequences of pre-stimulation warrant careful interpretation, particularly with respect to the feeding phenotypes observed across task contexts.

The NSF and fast-refeeding assays provide partially dissociable evidence for BNST CRF involvement in feeding regulation. Although feeding latency and thigmotaxis are spatially related in the NSF arena, the absence of a latency difference in the home cage — where avoidance demands are minimal — indicates that the NSF latency phenotype is not a general consequence of BNST CRF activation but emerges specifically at the conjunction of threat and feeding demand. Consistent with this interpretation, the fast-refeeding assay independently demonstrates that BNST CRF activation suppresses consummatory intake in a low-threat context, providing convergent evidence that BNST CRF circuitry amplifies the competition between avoidance and feeding under conditions of environmental challenge. Collectively, these acute findings establish BNST CRF neurons as a state-setting node capable of biasing behavior toward avoidance and away from feeding across multiple task contexts.

However, the acute timescale of these manipulations is more analogous to a discrete, bounded stress response than to the sustained CRF dysregulation that characterizes chronic stress-related psychiatric conditions (Musazzi et al., 2017; Yang et al., 2025). We therefore asked whether persistent activation of BNST CRF neurons would produce stable phenotypes consistent with a disease-relevant state (Montgomery et al., 2024). Strikingly, chronic NaCh-mediated activation of BNST CRF neurons recapitulated both the avoidance and hypophagic phenotypes observed following acute stimulation, consistent with the enduring behavioral sequelae of chronic stress-related circuit dysregulation that characterizes stress-related eating and anxiety disorders (Frank, 2021; Melles et al., 2021). Notably, BNST-targeted deep brain stimulation in refractory eating disorders has been reported to improve eating-related and affective symptoms (Manuelli et al., 2020; Blomstedt et al., 2017), consistent with a broader role for BNST activity in coordination of threat and feeding-related pathology. While our studies do demonstrate transient changes to body weight, normalization by day 30 suggests recruitment of compensatory homeostatic mechanisms that limit the impact of sustained BNST activation on long-term energy balance. Together, these findings motivated examination of the downstream projection targets through which BNST CRF neurons exert these behavioral effects.

We examined BNST CRF projections to the paraventricular hypothalamic nucleus (PVH) and lateral hypothalamus (LH), two major downstream GABAergic and CRFergic sensitive targets implicated in anxiety and feeding (Ly et al., 2025; Kim et al., 2015). This latter point is crucial given the main output signals of these neurons are both GABAergic and CRFergic (Dabrowska et al., 2013). The PVH has long been understood to be a major brain area that controls energy homeostasis and feeding (Leibowitz et al., 1981; Cowley et al., 1999; Balthasar et al., 2005). The lack of feeding effects from BNST CRF→PVH stimulation may reflect PVH circuit heterogeneity. By contrast, the lateral hypothalamus (LH) is another well-established feeding center, with lesions producing starvation and disrupted food seeking (Anand and Brobeck, 1951). Contemporary studies further show that targeted LH inhibition also leads to decreased feeding (Kovács et al., 2024). Hence, it is consistent that photostimulating BNST CRF neurons, which likely co-release both GABA and CRF, decrease food consumption. Similarly, the LH is known for controlling both motivational state as well as anxiety behaviors (Weera et al., 2023; Giardino et al., 2018; Owens-French et al., 2022; Liu et al., 2025). Interestingly, LH GABAergic input and signaling is also key to promoting anxiety behaviors (Wang et al., 2023; Guo et al., 2024). Thus, the avoidance-promoting effects of BNST CRF → LH terminal field stimulation are consistent with prior observations, although the targeted cell types remain unknown. Importantly, broad BNST GABAergic projections to the LH have been shown to promote feeding (Jennings et al., 2013a), an effect apparently opposite to that observed here. This apparent paradox may be resolved by the molecular and functional heterogeneity of BNST GABAergic neurons. We propose that whereas the GABAergic BNST CRF neurons preferentially engage LH circuits that mediate threat responses and hypophagia, broad GABAergic BNST populations engage LH circuits mediating appetitive behaviors. Such functional divergence could arise from differences in post-synaptic target identity, input specificity (Beier et al., 2015; Metz et al., 2021), or co-transmitting profiles (Dabrowska et al., 2013; Hnasko et al., 2010; Biglari et al., 2021). Distinguishing whether BNST CRF and non-CRF GABAergic neurons converge on overlapping or segregated LH cell populations remains an important question for future work. More broadly, BNST CRF subpopulations may differentially recruit downstream targets based on contextual environmental or internal need to generate an adaptive behavioral or physiologic response. The amygdala represents one such target of particular interest. Although we observed robust BNST CRF terminals in the amygdala (Figure 4A), we prioritized the LH and PVH for functional study given their well-established roles as primary substrates for integrating stress with feeding and avoidance. The amygdala, by contrast, shapes feeding in a predominantly cue- and context-dependent manner that enable learned cues to potentiate feeding even in sated states (Petrovich et al., 2002), while central amygdala circuits mediate the suppression of feeding by aversive cues (Petrovich et al., 2009). Given this capacity to align feeding with environmental and emotional context, the BNST CRF→amygdala projection represents a compelling substrate through which threat-related states may further refine feeding behavior, and an important direction for future investigation.

### 4.3 Limitations

A key limitation is that our manipulations do not isolate CRF ligand–receptor signaling, leaving the relative contributions of peptide release versus fast neurotransmission unresolved. Nonetheless, CRF signaling likely contributes, as BNST CRF receptor antagonism ameliorates stress-induced binge eating (Micioni Di Bonaventura et al., 2014). This demonstrates that CRF receptor signaling in the BNST gates stress-evoked feeding. In other CRF circuits, the anxiety-like effects of activating CRF neurons depend on their level of activity, previous exposure to chronic stress, and baseline levels of anxiety (Pomrenze et al., 2019; Ugolini et al., 2008; Sandi et al., 2008).

A further limitation concerns the pre-stimulation optogenetic paradigm employed here. While BNST CRF neuron activity returned to baseline within approximately five minutes of stressor offset in our photometry recordings, the duration of the behavioral state shift induced by optogenetic pre-stimulation was not independently characterized across defined post-stimulation intervals. It therefore remains unclear whether the avoidance or hypophagic phenotype observed in ChR2-expressing mice persists for seconds, minutes, or longer following stimulation offset. Future time-course behavioral experiments will be necessary to fully characterize the temporal dynamics of BNST CRF-driven behavioral state changes.

An additional limitation concerns the interpretation of terminal field stimulation experiments. Axon terminal photostimulation can antidromically activate cell bodies, which may in turn engage collateral projections beyond the targeted site. The behavioral dissociation between LH and PVH terminal stimulation provides evidence that the LH represents a preferential effector locus, but does not fully exclude contributions from antidromically activated collaterals. Future experiments employing complementary approaches — such as soma-targeted inhibition during terminal stimulation — will be necessary to establish strictly pathway-restricted conclusions (Hsu et al., 2025). An important interpretive caveat is that although BNST CRF activation suppresses feeding, we cannot fully distinguish a primary feeding output from a secondary consequence of elevated threat/avoidance. This ambiguity extends to our photometry recordings, where the absence of a non-food object control leaves open whether the approach-locked BNST CRF activity observed during novelty-suppressed feeding reflects conflict-specific engagement or a more generalized response to novelty or object approach. Still, several features argue against non-specific stress-induced hypophagia. First, the effect is only generated with BNST CRF→LH terminal stimulation (absent with PVH stimulation despite its role in stress-axis regulation (Qin et al., 2018). Second, LH circuitry exhibits the opposite organization, wherein metabolic signals such as leptin regulate LH-driven HPA activation and corticosterone release, demonstrating that metabolic need can shape stress responses (Bonnavion et al., 2016, 2015). Third, BNST CRF activation does not uniformly change feeding across all contexts: feeding latency in the home cage was unaffected, whereas feeding in anxiogenic or energetically demanding contexts was suppressed. This context-dependence suggests that BNST CRF neurons gate feeding based on environmental threat level rather than simply inducing a non-specific hypophagic state.

Finally, although both male and female mice were included across all experiments, the present study was not powered to systematically detect sex-dependent differences in BNST CRF circuit function, and data were pooled across sexes. Given evidence for sex-specific functional properties of BNST CRF neurons in related contexts (Yu et al., 2021), as well as the disproportionate prevalence of anxiety and stress-related eating disorders in females (Smink et al., 2012), future studies explicitly powered to examine sex as a biological variable in this circuit are warranted.

## Data Availability

The raw data supporting the conclusions of this article will be made available by the authors, without undue reservation.

## Supplementary material

Supplementary Extended Data Table 1 Summaryof statistical analyses for all experiments and all main figures. This table provides a consolidated summary of all statistical tests associated with the main figures. Each row reports the corresponding figure and panel, outcome measure, comparison groups, statistical test, test statistic, degrees of freedom (df), sample sizes (N), exact p-value, and significance level. Photometry data (Figure 1) were analyzed using two- tailed one- sample t-tests against a normalized baseline of 0. Behavioral and physiological comparisons between two groups were analyzed using two-tailed Welch’s unpaired t-tests unless otherwise indicated. Non-parametric comparisons (Figure 4, center- time measures) were analyzed using two- tailed Mann–Whitney U tests. Repeated- measures data (Figure 3G) were analyzed using two- way repeated- measures ANOVA. Normality was assessed within each group using the Shapiro–Wilk test prior to statistical testing to guide the choice of parametric versus non-parametric analyses. Degrees of freedom are reported as integers for one- sample t-tests and as fractional values for Welch’s t-tests, reflecting the Welch–Satterthwaite approximation. For ANOVA, degrees of freedom are reported as numerator/denominator pairs. Sample sizes (N) indicate the number of animals per group; for one- sample tests, N reflects the number of animals compared to baseline. “—” denotes not applicable. All statistical tests were two- tailed. Significance is denoted as follows: ns, not significant (*p* ≥ 0.05); *p* < 0.05; *p* < 0.01.

Supplementary Extended Data Figure 1-1 The 405 nm isosbestic control channel remains stable across recording epochs and behavioral paradigms. **(A,B)** Mean 405 nm isosbestic signal amplitude during baseline (black), stressor exposure (pink), and post-stimulus periods (teal) for forced swim stress **(A)** and predator odor **(B)** recordings. Signals remain stable within each epoch, with no systematic stimulus-evoked drift. A transient step-down at stressor onset in panel **(A)** reflects the expected motion artifact associated with physical entry into the swim apparatus, which is corrected by the regression-based normalization procedure. **(C)** Event-locked 405 nm isosbestic traces aligned to approach (black) and bite (pink) events during novelty-suppressed feeding. The absence of systematic deflection at event onset (t = 0) confirms that event-locked ΔF/F responses in the 465 nm channel reflect calcium-dependent neural activity rather than motion artifacts. Traces are presented as mean ± SEM. Individual animal traces shown in lighter shading. All signals reflect 2 Hz low-pass filtered raw fluorescence prior to regression-based normalization.

Supplementary Extended Data Figure 2-1 NaCh expression increases excitability and alters firing patterns in BNST CRF neurons. **(A)** Resting membrane potential of control and NaCh-expressing BNST CRF neurons. **(B)** Action potential threshold of control and NaCh-expressing BNST CRF neurons. **(C)** Distribution of spontaneous firing patterns (silent, single action potential, or burst firing) in control and NaCh-expressing neurons. **(D)** Representative whole-cell current-clamp traces from a control neuron (top) exhibiting narrow, rapidly repolarizing action potentials, and a NaCh-expressing neuron (bottom) exhibiting characteristically broadened action potentials with a prolonged depolarizing plateau and slow repolarization (half-maximal width: 0.6–1.2 s). Box-and-whisker plots show the median (center line), interquartile range (box), and minimum–maximum values (whiskers); each point represents one cell. Data were obtained from 2 animals per group and are presented as functional validation of NaCh expression rather than as fully powered statistical comparisons. **(A,B)** Membrane properties were analyzed using two-tailed Welch’s unpaired *t*-tests: **(A)** resting membrane potential, *p* = 0.1632; **(B)** action potential threshold, *p* = 0.0067. **(C)** Firing pattern distributions are presented descriptively. Significance: ***p* < 0.01.

Supplementary Extended Data Figure 3-1 Residual analysis validating energy expenditure estimates derived from indirect calorimetry. Scatter plot showing residuals from a linear regression relating computed energy balance to change in adipose tissue mass across individual mice. Energy balance was calculated from measured energy intake and indirect calorimetry–derived energy expenditure, while adipose tissue change was quantified by pre and post calorimetry body composition measurements. Because changes in adipose mass are expected to reflect cumulative energy balance, concordance between these measures provides an internal validation of the derived energy expenditure values. Residuals are plotted as a function of adipose tissue change, with each point representing one mouse (control, black; NaCh, pink). One data point exhibited a large negative residual, corresponding to a z scored residual of 2.75, indicating that the observed energy balance substantially deviated from that predicted by adipose tissue loss. This point was flagged as a potential outlier for data quality assessment but was retained in all analyses, as its exclusion did not alter the interpretation of any results. Mean residual = 6.908 × 10–16, Standard deviation of residual: 2.494.
