# Supplementary figures and images for: Corticotropin-releasing factor neurons in the bed nucleus of the stria terminalis modulate avoidance behaviors and feeding
Source: Front Neurosci. 2026 Jul 20;20:1876369. doi: 10.3389/fnins.2026.1876369 (PMC13430618; doi:10.3389/fnins.2026.1876369)

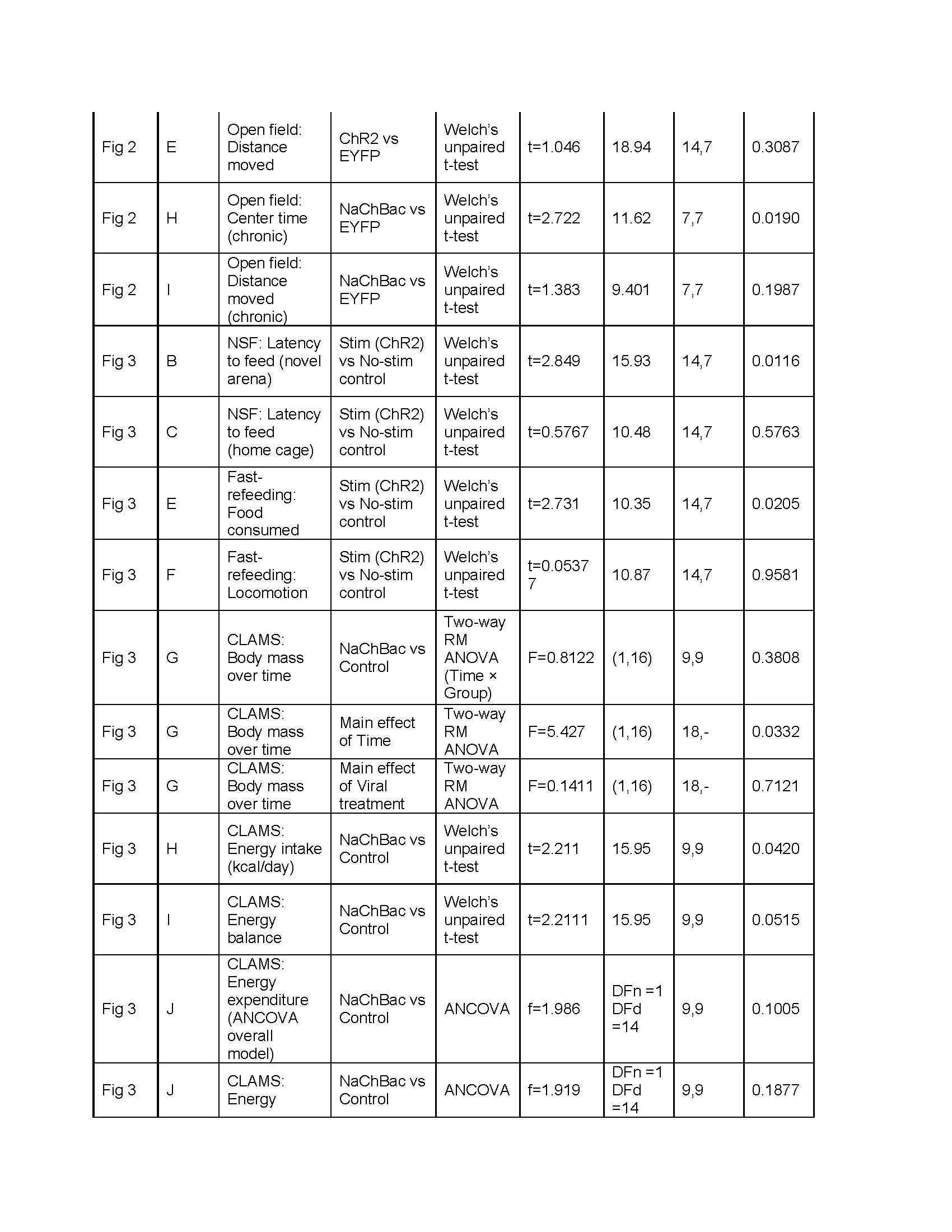

Supplement: Supplementary file 1 [file Image_3.tif]

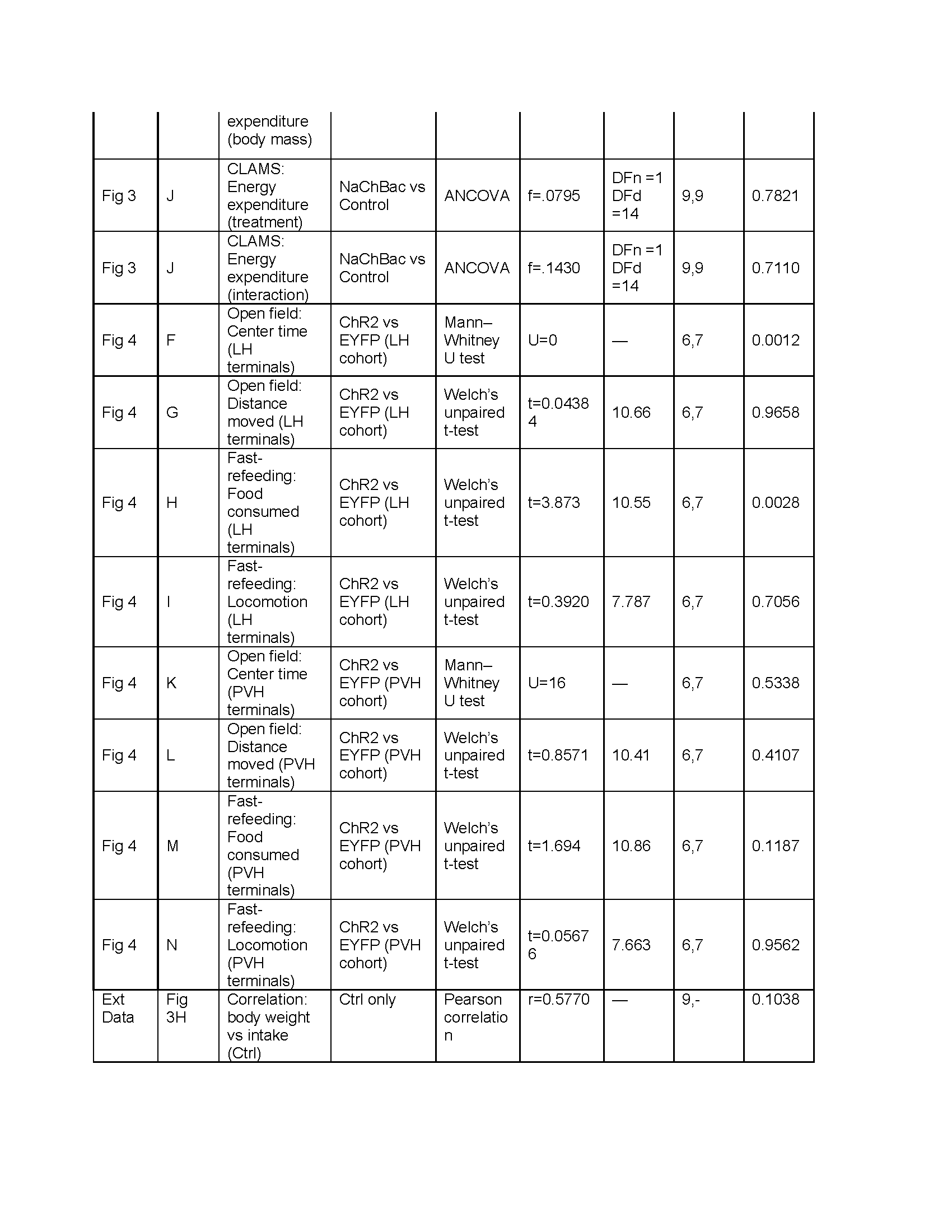

Supplement: Supplementary file 2 [file Image_4.tif]

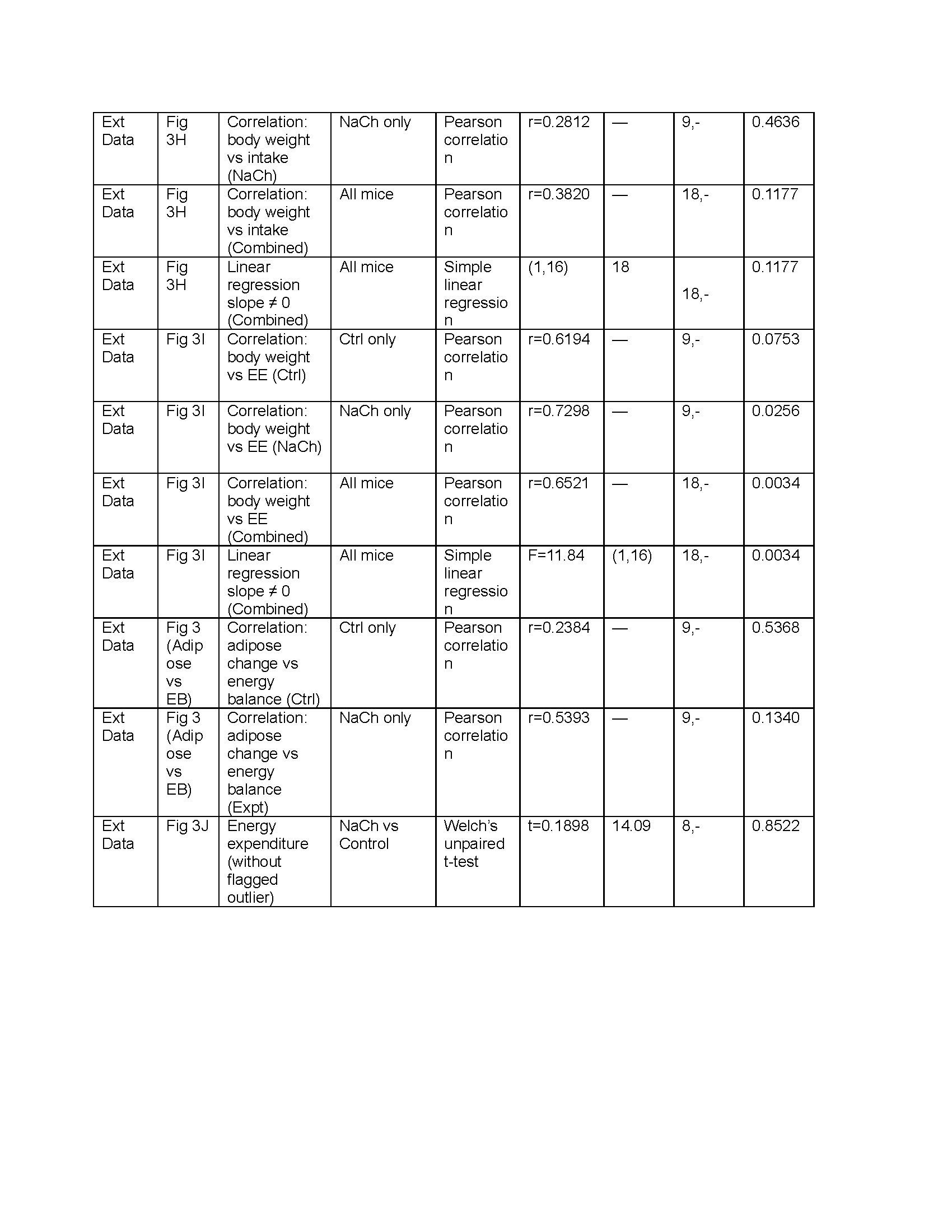

Supplement: Supplementary file 3 [file Image_5.tif]

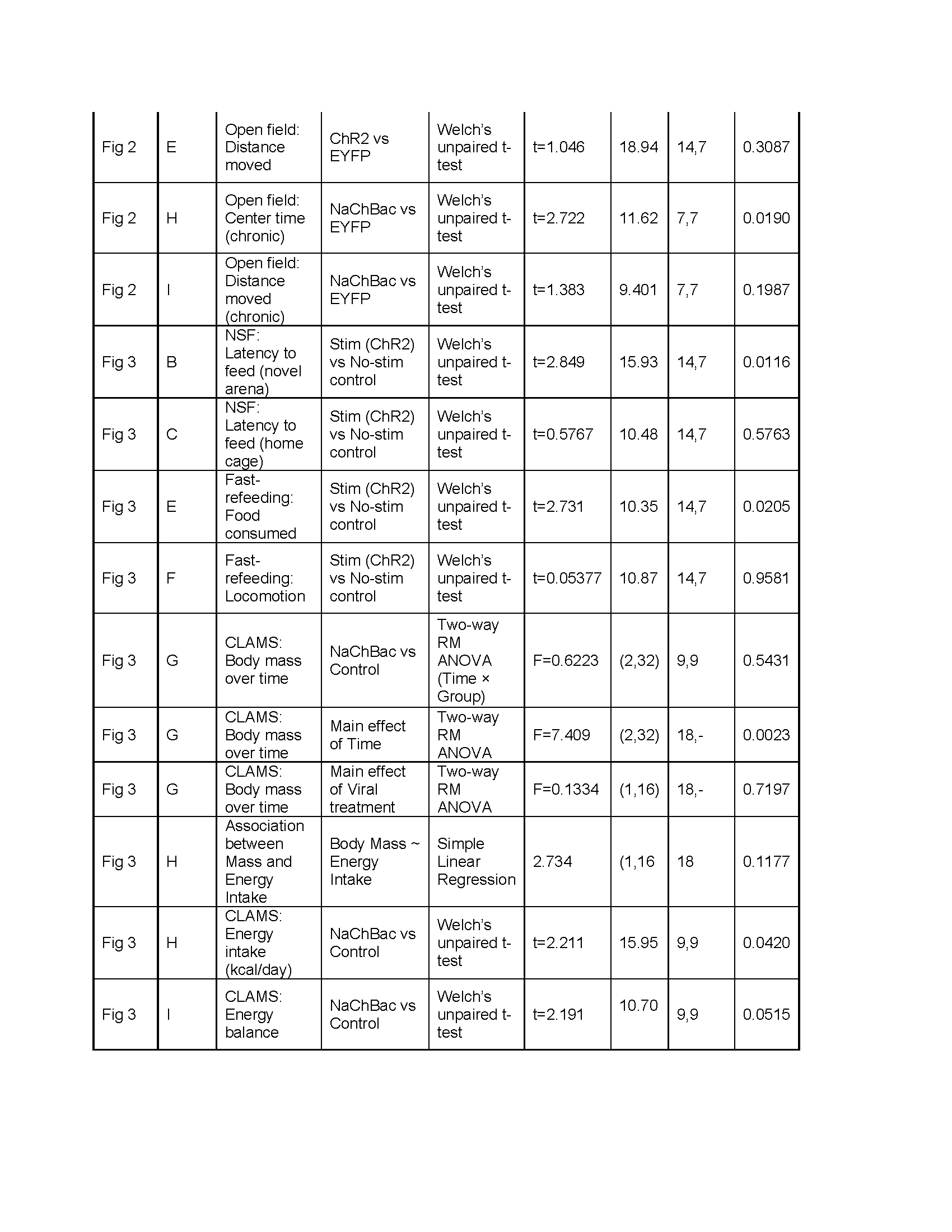

Supplement: Supplementary file 4 [file Image_6.tif]

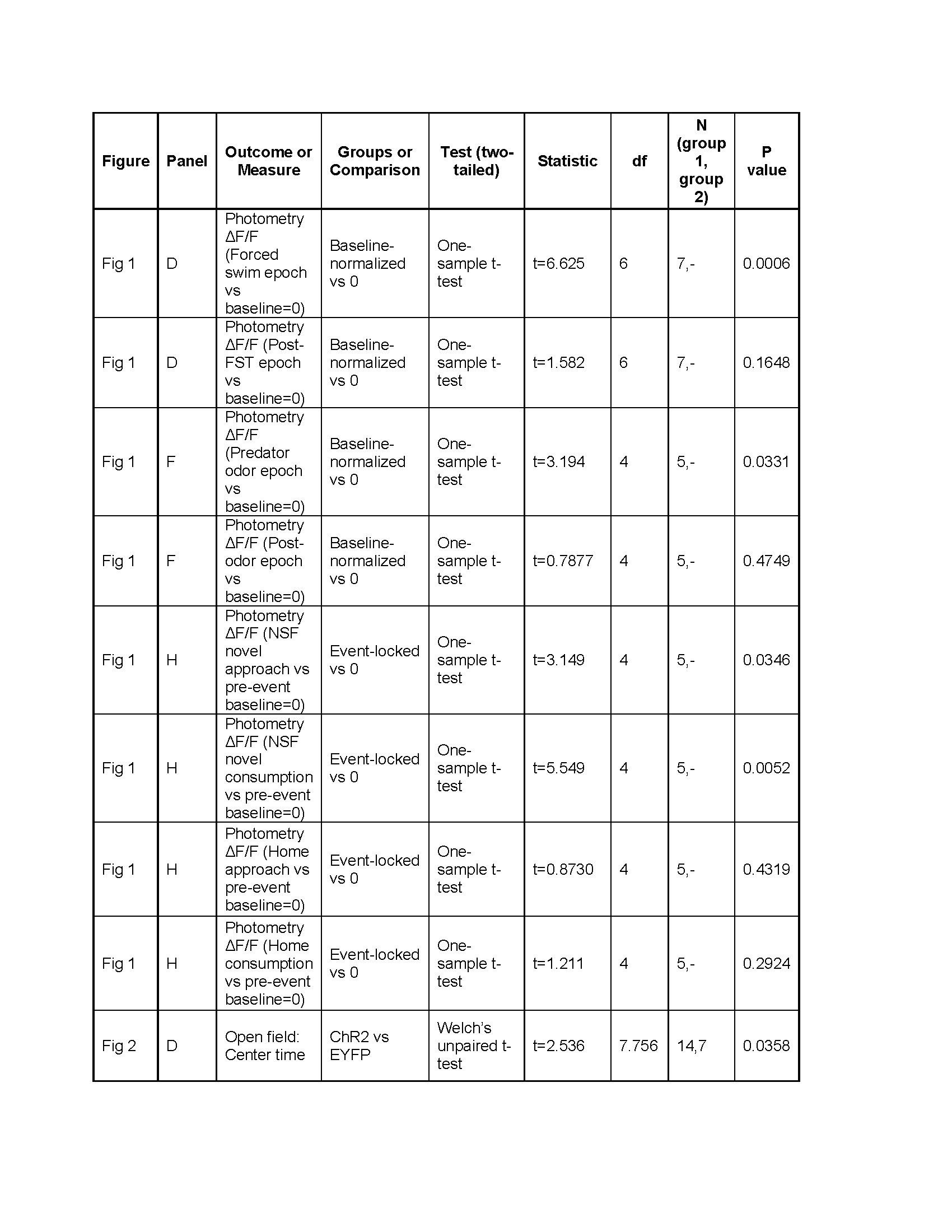

Supplement: Supplementary file 5 [file Image_8.tif]

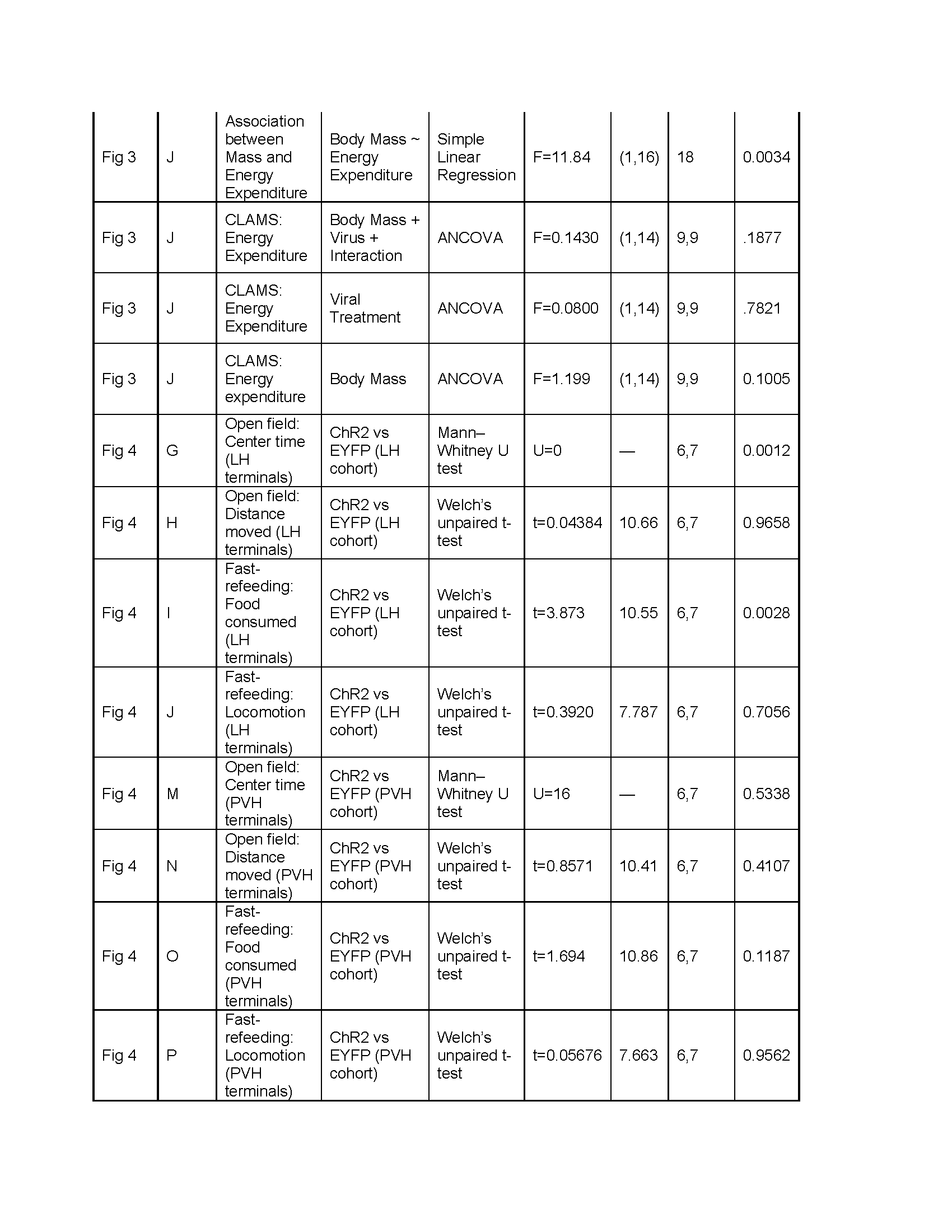

Supplement: Supplementary file 6 [file Image_9.tif]

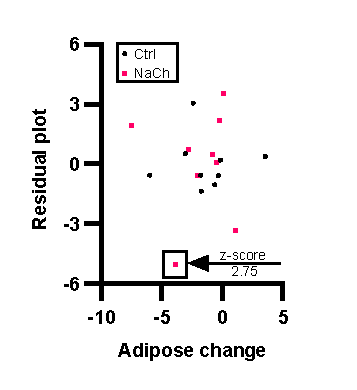

Supplement: Supplementary file 7 [file Image_11.tif]

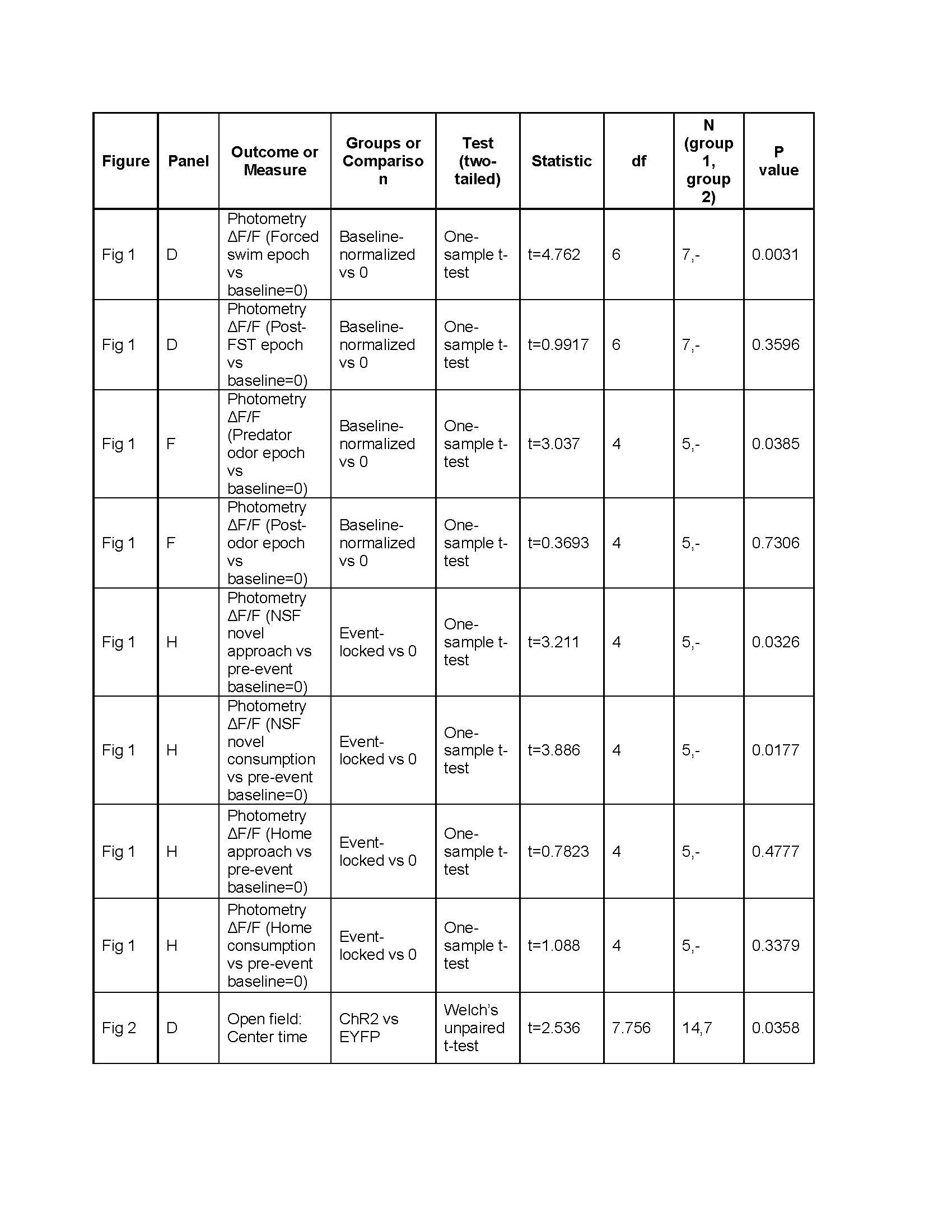

Supplement: Supplementary Extended Data Table 1 — Summaryof statistical analyses for all experiments and all main figures. This table provides a consolidated summary of all statistical tests associated with the main figures. Each row reports the corresponding figure and panel, outcome measure, comparison groups, statistical test, test statistic, degrees of freedom (df), sample sizes (N), exact p-value, and significance level. Photometry data (Figure 1) were analyzed using two- tailed one- sample t-tests against a normalized baseline of 0. Behavioral and physiological comparisons between two groups were analyzed using two-tailed Welch’s unpaired t-tests unless otherwise indicated. Non-parametric comparisons (Figure 4, center- time measures) were analyzed using two- tailed Mann–Whitney U tests. Repeated- measures data (Figure 3G) were analyzed using two- way repeated- measures ANOVA. Normality was assessed within each group using the Shapiro–Wilk test prior to statistical testing to guide the choice of parametric versus non-parametric analyses. Degrees of freedom are reported as integers for one- sample t-tests and as fractional values for Welch’s t-tests, reflecting the Welch–Satterthwaite approximation. For ANOVA, degrees of freedom are reported as numerator/denominator pairs. Sample sizes (N) indicate the number of animals per group; for one- sample tests, N reflects the number of animals compared to baseline. “—” denotes not applicable. All statistical tests were two- tailed. Significance is denoted as follows: ns, not significant (p ≥ 0.05); p < 0.05; p < 0.01. [file Image_2.tif]

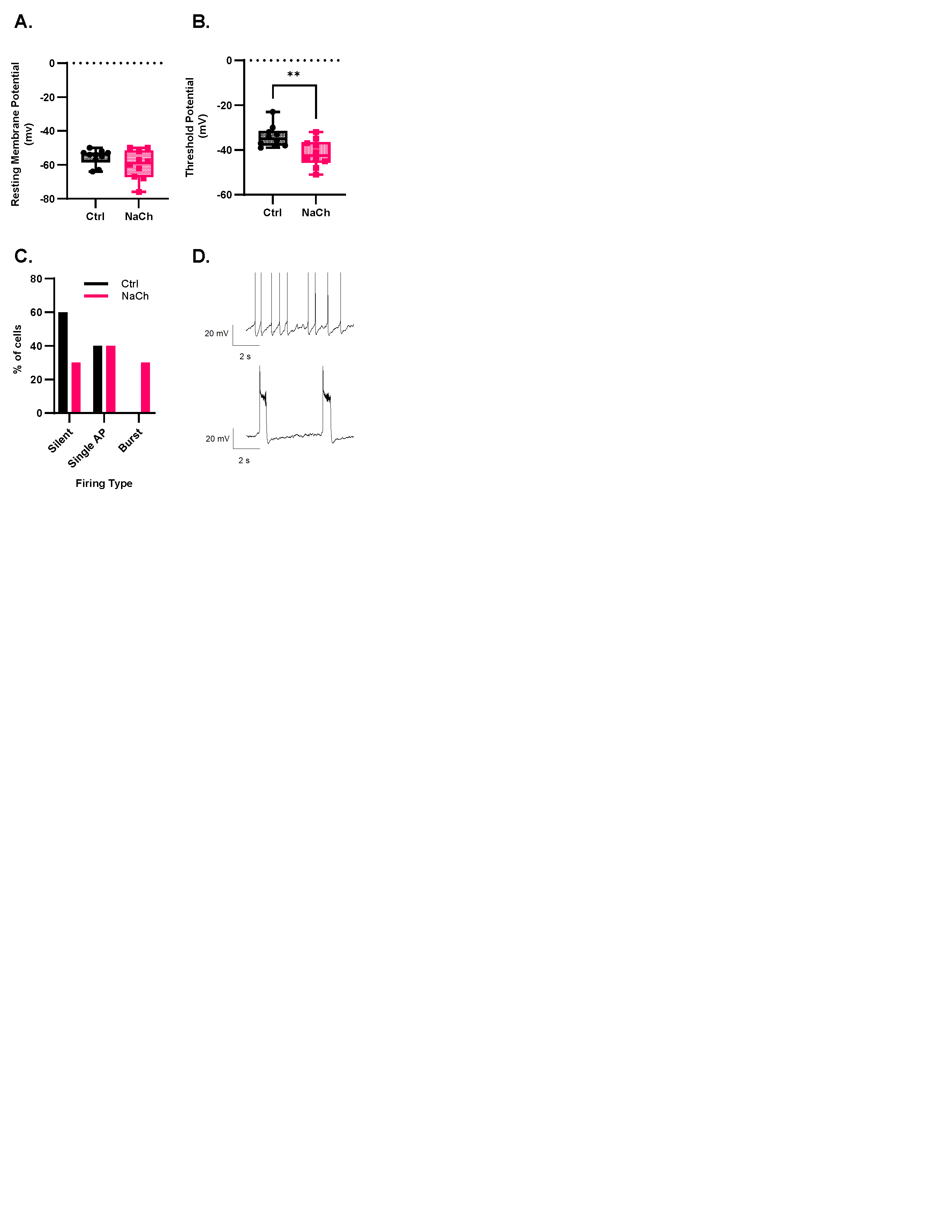

Supplement: Supplementary Extended Data Figure 2-1 — NaCh expression increases excitability and alters firing patterns in BNST CRF neurons. (A) Resting membrane potential of control and NaCh-expressing BNST CRF neurons. (B) Action potential threshold of control and NaCh-expressing BNST CRF neurons. (C) Distribution of spontaneous firing patterns (silent, single action potential, or burst firing) in control and NaCh-expressing neurons. (D) Representative whole-cell current-clamp traces from a control neuron (top) exhibiting narrow, rapidly repolarizing action potentials, and a NaCh-expressing neuron (bottom) exhibiting characteristically broadened action potentials with a prolonged depolarizing plateau and slow repolarization (half-maximal width: 0.6–1.2 s). Box-and-whisker plots show the median (center line), interquartile range (box), and minimum–maximum values (whiskers); each point represents one cell. Data were obtained from 2 animals per group and are presented as functional validation of NaCh expression rather than as fully powered statistical comparisons. (A,B) Membrane properties were analyzed using two-tailed Welch’s unpaired t-tests: (A) resting membrane potential, p = 0.1632; (B) action potential threshold, p = 0.0067. (C) Firing pattern distributions are presented descriptively. Significance: **p < 0.01. [file Image_7.tif]

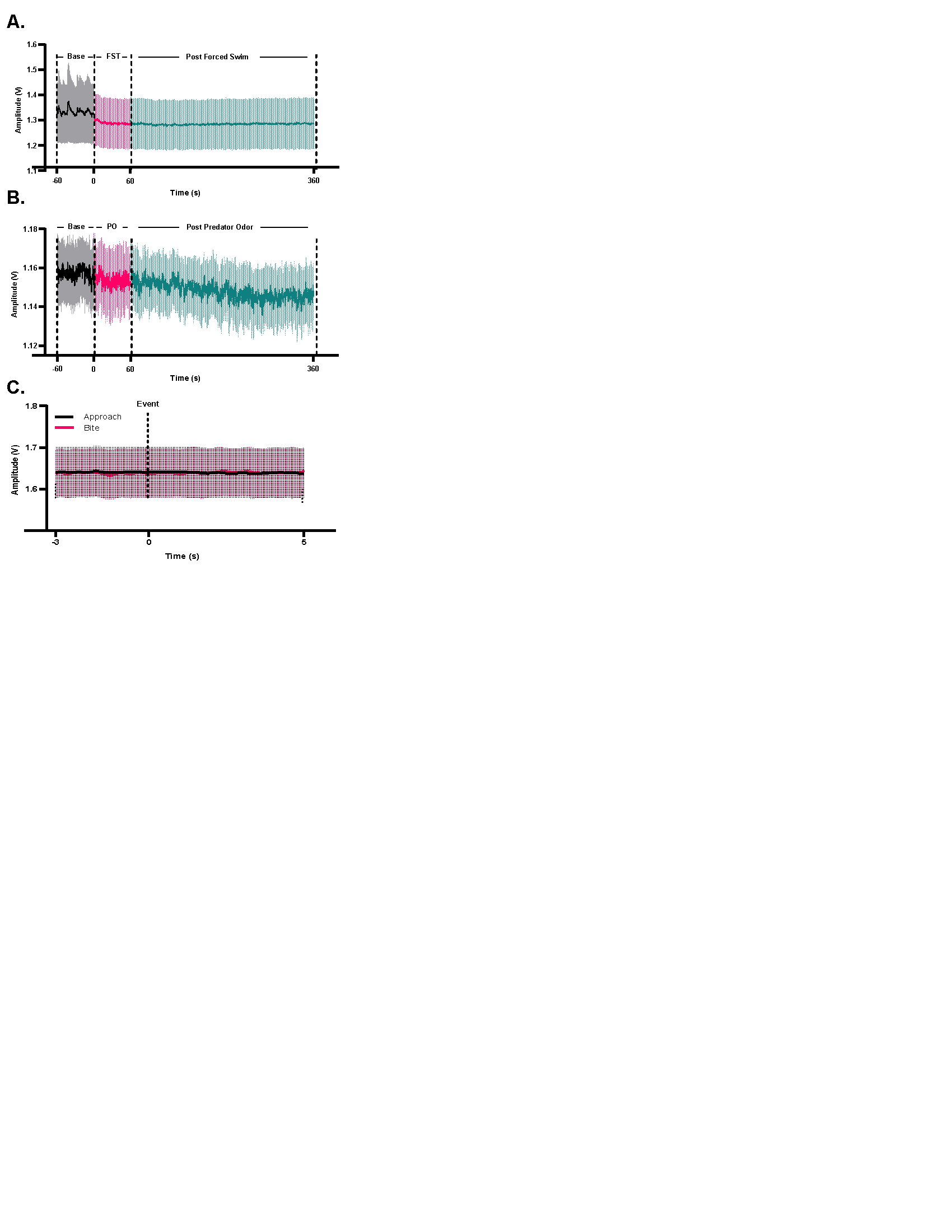

Supplement: Supplementary Extended Data Figure 3-1 — Residual analysis validating energy expenditure estimates derived from indirect calorimetry. Scatter plot showing residuals from a linear regression relating computed energy balance to change in adipose tissue mass across individual mice. Energy balance was calculated from measured energy intake and indirect calorimetry–derived energy expenditure, while adipose tissue change was quantified by pre and post calorimetry body composition measurements. Because changes in adipose mass are expected to reflect cumulative energy balance, concordance between these measures provides an internal validation of the derived energy expenditure values. Residuals are plotted as a function of adipose tissue change, with each point representing one mouse (control, black; NaCh, pink). One data point exhibited a large negative residual, corresponding to a z scored residual of 2.75, indicating that the observed energy balance substantially deviated from that predicted by adipose tissue loss. This point was flagged as a potential outlier for data quality assessment but was retained in all analyses, as its exclusion did not alter the interpretation of any results. Mean residual = 6.908 × 10^–16^, Standard deviation of residual: 2.494. [file Image_10.tif]
